# Kirigami-based metastructures with programmable multistability

**DOI:** 10.1073/pnas.2117649119

**Published:** 2022-03-07

**Authors:** Xiao Zhang, Jiayao Ma, Mengyue Li, Zhong You, Xiaoyan Wang, Yu Luo, Kaixue Ma, Yan Chen

**Affiliations:** ^a^School of Mechanical Engineering, Tianjin University, Tianjin 300072, China;; ^b^Department of Engineering Science, University of Oxford, Oxford OX1 3PJ, United Kingdom;; ^c^The Tianjin Key Laboratory of Imaging and Sensing Microelectronic Technology, School of Microelectronics, Tianjin University, Tianjin 300072, China;; ^d^Key Laboratory of Mechanism Theory and Equipment Design of Ministry of Education, Tianjin University, Tianjin 300072, China

**Keywords:** kirigami cuboid, multistability, metastructure, programmability

## Abstract

Multistability plays an important role in advanced engineering applications such as metastructures, deployable structures, and reconfigurable robotics. However, most existing multistability design is based on the two-dimensional (2D)/3D series or parallel combinations of bistable unit cells, which are derived from snap-through instability, nonrigid foldable origami structures, and compliant mechanism, due to the lack of a generic multistable unit cell. Here, we develop a tristable kirigami cuboid by creating a set of elastic joints only effective in a specific motion range which integrates the elastic sheets and switchable hinge axes inspired by the kinematic behaviors of a kirigami cuboid with thick facets. The energy barriers between the stable states can be programmed by the geometric design parameters and material properties of the elastic joints. Taking the tristable cuboid as a unit cell, we construct a family of metastructures with multiple stable states. The number of stable states, the combination of unit stable states, and their transform sequences can be programmed by the number of unit cells, unit design parameters, and loading modes and loading sequences. We also apply this tristable cuboid to the design of frequency reconfigurable antenna with three programmable working frequencies, which demonstrates that such versatile multistability and structural diversity facilitate the development of multifunctional materials and devices.

## Significance

Different from most existing multistable structures whose multiple stable states are achieved through the combinational effect of bistable units, we invent a generic tristable kirigami cuboid. The three stable states have fundamentally distinct geometric configurations and chirality, and the transformation among them can be realized by tension/compression or clockwise/counterclockwise twist. Tessellating the units in series, a family of multistable metamaterials can be constructed, the mechanical behaviors of which are programmable by the unit geometry, the material of the elastic joints, the number of units, and the loading conditions. As a demonstration of the potential applications, a frequency reconfigurable antenna for 5G triple-band communication is developed based on a tristable unit, and the frequency tunability is verified by experiments.

Multistability is a characteristic of structures with more than one stable equilibrium configuration, which can realize the rapid structural reconfiguration to meet certain functional requirements. Recently, multistable structures have been used to design mechanical structural materials with shape reconfiguration (1–3) and negative stiffness (4) for trapping elastic strain energy (5), energy absorption (6–8), and ternary logic operation (9); robots (10–14) for simplifying actuators, reducing power consumption, and improving the locomotion speed and motion integration; soft media (15) and mechanical diodes (16, 17) for the propagation of mechanical signals; devices for mechanical memory storage (18); deployable structures for self-locked configuration (19, 20) and rapid deployment (21); and other potential applications (22).

However, most existing multistability is based on the two-dimensional (2D)/3D series or parallel combinations of bistable unit cells, which are derived from snap-through instability (1, 8, 17, 23–27), nonrigid foldable origami structures (28–32), and compliant mechanisms including rigid origami (33–37). Among them, the snap-through instable beam or structure is the most commonly used fundamental unit in construction with planer motifs or spatial topologies to form 1D, 2D, and 3D multistable structures with unidirectional (1, 24), bidirectional, and multidirectional multistability (2, 4, 6, 9), such as the multistable 1D cylindrical structures, 2D square lattices, and 3D cubic/octahedral lattices (9). Recently, nonrigid origami structure is an emerging resource for designing bistable units based on the elastic deformation of origami facets, such as the Kresling pattern (11, 18, 20, 38) and the hypar pattern (30, 39). Multiple Kresling units can be assembled in series to construct multistable structures (18, 31), and multiple hypar-origami units can be tessellated in plane to be a multistable metasurface (30). Meanwhile, compliant mechanisms derived from mechanisms by introducing spring hinges with compliant segments (34, 36) or torsional springs (40) to store energy have been used to propose bistable unit cells, such as four-bar developable mechanisms (37), Sarrus linkages (41), twisting and rotational mechanisms (42, 43), rotating polygon embedded magnets (44–46), the waterbomb unit (34), and the Miura-ori unit (47, 48). The Miura-ori units have been stacked to be multilayer multistable structures (16, 33, 47).

Besides few tristable units with nonzero energy stable states (32, 41), there is no generic tristable or multistable structure which itself is a basic unit rather than constructing with bistable units. On the other hand, most of the bistable unit cells are accompanied by large deformation on beams or facets, while few are derived from the design of joints. One such example is quadrastable overconstrained spatial Sarrus mechanisms with compliant joints (41), whose stable states are also nonzero energy ones, except the initial fabrication state. Therefore, in this paper, we are aiming to develop a generic tristable kirigami cuboid with a set of specially designed elastic joints based on its kinematic behaviors. By combining the tristable kirigami cuboid in series, multistable structures with programmable stable configurations, transformation sequence, and stiffness are constructed. This work paves the way to design multistable metastructures, which facilitates the development of functional materials and devices.

## Results

### The Kirigami-Inspired Foldable Cuboid.

**Fig. 1.**
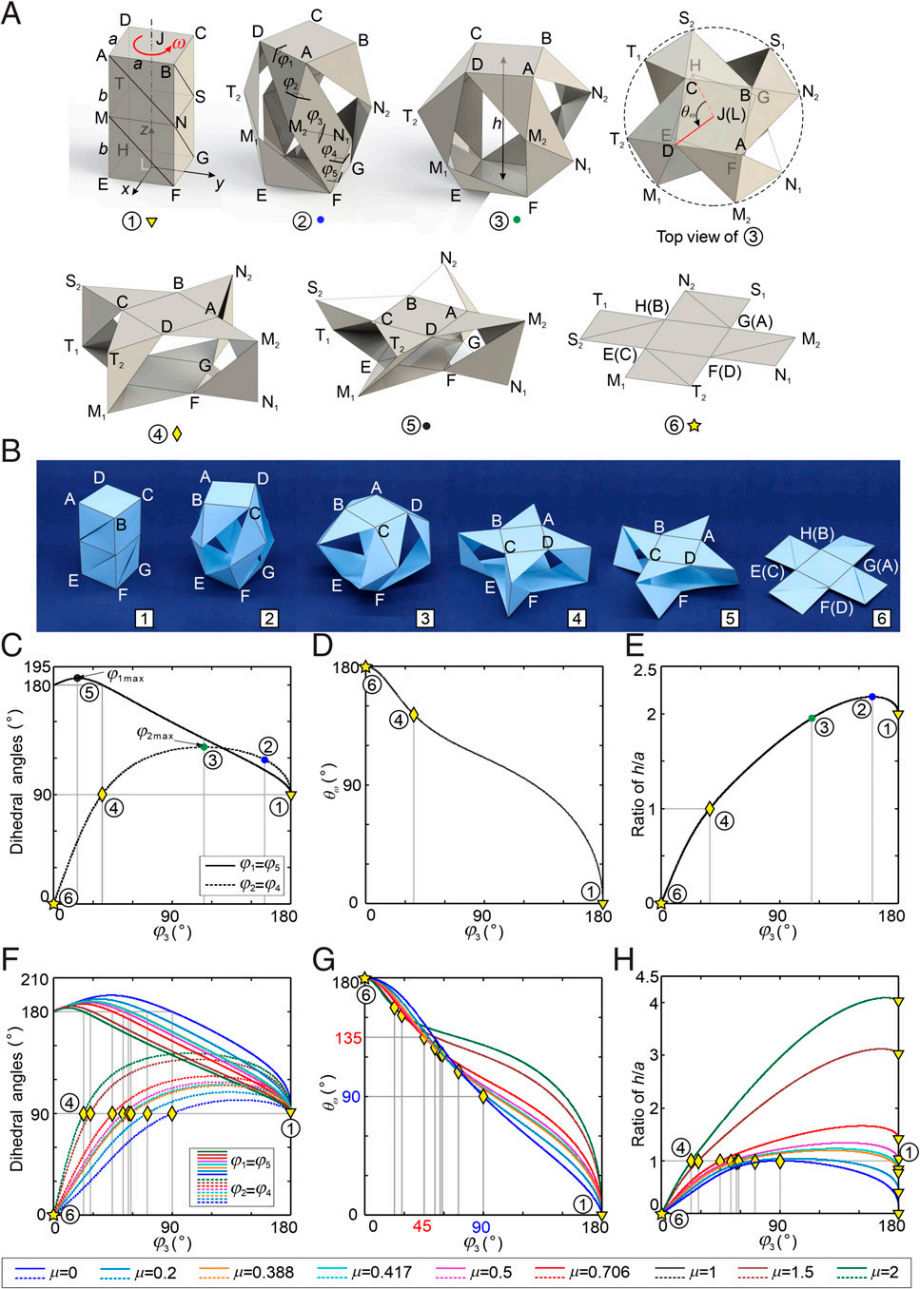
Geometry and kinematics of the foldable kirigami cuboid. (*A*) Folding sequence of the left-handed kirigami cuboid with sizes *b = a* (length ratio μ = *b/a* = 1), where ① is the closed cuboid, ② is the configuration with the maximum distance *h* between the top facet and bottom facet, ③ is the configuration with maximum dihedral angles φ_2_ and φ_4_, ④ is an opened configuration with φ_1_ = φ_5_ = 180°, φ_2_ = φ_4_ = 90°, ⑤ is the configuration with maximum dihedral angles φ_1_ = φ_5_ > 180°, and ⑥ is the flat configuration with φ_1_ = φ_5_ = 180° and φ_2_ = φ_3_ = φ_4_ = 0°. Here, the top view of ③ shows the twist angle *θ_ω_* which is measured from LH to JD along the positive direction of the *z* axis to show the rotation angle of the top facet relative to the bottom facet from ① to ⑥. (*B*) Folding sequence of the right-handed kirigami cuboid. (*C*–*E*) The dihedral angles of *φ_i_* (*I =* 1, 2, 4, 5), the twist angle *θ_ω_* and the height ratios of *h/a* vs. *φ*_3_, where ①–⑥ corresponds to the configurations in *A* with μ = 1. (*F*–*H*) The geometry and kinematics of the kirigami cuboid with ratio μ = 0, 0.2, 0.388, 0.417, 0.5, 0.706, 1.5, and 2.

**Fig. 2.**
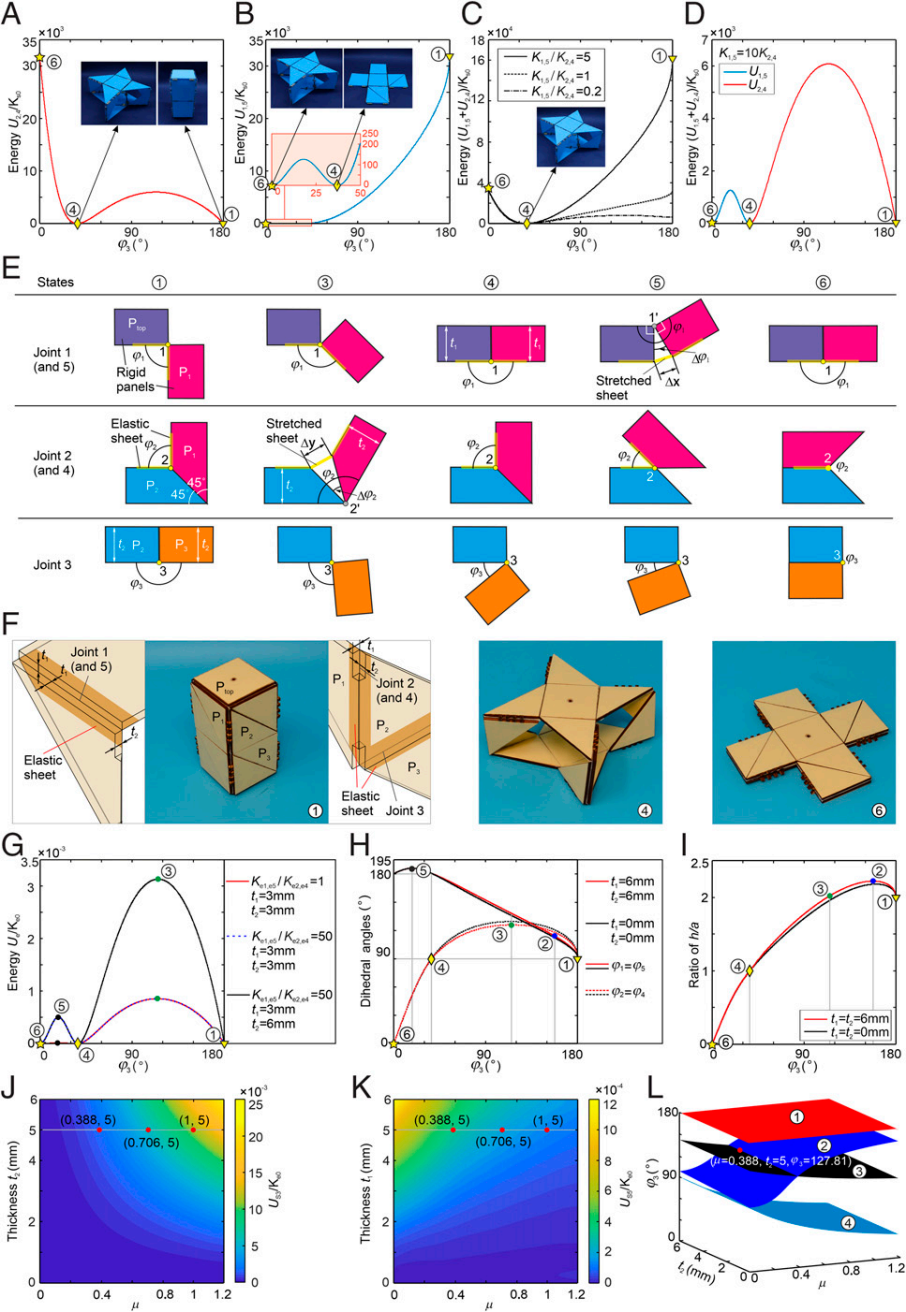
The construction of the tristable structure and the programmability of the energy barriers. (*A*) The energy *U*_2,4_ of the system when installing torsional springs with rest angles φ_20_ = φ_40_ = 90° and stiffness *K*_2,4_ at hinges 2 and 4 in all limbs to form a bistable structure with stable states ① and ④. (*B*) The energy *U*_1,5_ of the system when installing torsional springs with rest angles φ_10_ = φ_50_ = 180° and stiffness *K*_1,5_ = *K*_2,4_ to hinges 1 and 5 in all limbs to form a bistable structure with stable states ④ and ⑥. (*C*) The energy of the system when installing the above two types of torsional springs to the corresponding hinges with only one stable state ④ under *K*_1,5_/*K*_2,4_ = 5, 1, 0.2. Here, *A*–*C*, *Insets* are corresponding prototypes. (*D*) The total energy of two types of torsional springs in all limbs (*U*_1,5_ + *U*_2,4_), where torsional springs with stiffness *K*_2,4_ are valid from the stable state ① to state ④ (i.e., *U*_2,4_ > 0 and *U*_1,5_ = 0), and the torsional springs with stiffness *K*_1,5_ (*K*_1,5_/*K*_2,4_ = 10) are valid from the stable state ④ to state ⑥ (i.e., *U*_2,4_ = 0 and *U*_1,5_ > 0), which leads to the tristable structure with three stable states ①, ④, and ⑥. (*E*) The elastic joints with different working functions at joints 1/5, 2/4, and 3 for different states. (*F*) The three stable states ①, ④, and ⑥ of the tristable wood structure with elastic joints. (*G*) The energy of the tristable structure based on elastic joints is tuned by varying the ratio *K*_e1,e5_/*K*_e2,e4_ and thickness of the panels. (*H* and *I*) The variations of dihedral angles and the height of the tristable structure constructed with elastic joints proposed in *E*. (*J* and *K*) The energy of tristable structures *U*_S3_ and *U*_S5_ in states ③ and ⑤, respectively, and the relationship between the energy barriers and the length ratio μ, thickness of panels. (*L*) The angles φ_3_ of states ①, ②, ③, and ④ under different μ and thickness of panels, which indicates state ② is between ① and ③ with μ > 0.388 and between ③ and ④ with μ < 0.388; when μ = 0.388, state ② and state ③ are coincident, when the panel thickness is 5 mm. Here, constants K_s0_ = 3.21 N.mm/rad, K_e0_ = *K*_ep0.3_*a*^3^ = 0.1037 × 80^3^ = 53,094 N.mm, where *K*_ep0.3_ (the per length [in millimeters] stiffness of the elastic sheet with thickness 0.3 mm) is derived from an experiment (*SI Appendix*, section 3).

We start from a square cuboid with eight vertices noted by A to H as shown in Fig. 1*A*, ①. Here, top facet ABCD and bottom facet EFGH are identical squares of side length *a*, and four side facets are rectangles of height 2*b*. The cuboid is a structure without mobility, even though we regard the edges of the cuboid as hinges. In order to make the cuboid foldable, first, we add four horizontal creases, MN, NS, ST, and TM, to the middle of the rectangular facets on the cuboid, which subsequently divide each facet into two rectangles of dimension *a* × *b*, in which we define μ = *b/a*; second, we cut each of these rectangles along the diagonal from top left to bottom right corners, which makes the top and bottom facets of the cuboid connected by four limbs, each composed with four triangle pieces jointed by the creases; that is, one limb is △DAM_2_–△AM_2_N_1_–△M_2_N_1_F–△N_1_FG, and there are five creases (joints), DA, AM_2_, M_2_N_1_, N_1_F, and FG, to connect with two square facets ABCD and EFGH (Fig. 1*A*, ②). In such a way, the cuboid is transformed into a foldable kirigami cuboid whose folding sequence is shown in Fig. 1*A*, ① to ⑥, when we twist the square ABCD counterclockwise from the top view, or the deployable sequence from Fig. 1*A*, ⑥ to ①, by twisting ABCD clockwise. Hence, we call the foldable cuboid in Fig. 1*A* left-handed. Alternatively, if we cut the eight rectangles along the diagonal from top right to bottom left corners, a right-handed foldable cuboid will be obtained (Fig. 1*B* and Movie S1).

No matter which chirality, the foldable cuboid is of multiple degrees of freedom if we take facets as rigid links and take creases as revolute joints. Here, we only consider the folding process such that the top facet ABCD is always parallel to the bottom one, EFGH, which kinematically requests that the four limbs are kept in a rotational symmetry about the *z* axis of this cuboid. Hence, the creases at the same position on the four limbs are synchronized. With this extra constraint, we can find the relationship among the dihedral angles along one limb, *φ_i_* (*i* = 1, 2, …, 5) as marked in Fig.1*A*, ② (*SI Appendix*, section 1). The relationships among the dihedral angles are

[1] $$\cos^{2}\varphi_{2}-\left(1+{\text{tan}}^{2}\varphi_{1}\right)\cos\;\varphi_{2}-\sin\;\varphi_{2}\tan\;\varphi_{1}\left(\sin\;\varphi_{1}\tan\;\varphi_{1}-2\mu +\cos\;\varphi_{1}\right)+\tan^{2}\varphi_{1}=0,\text{tan}\frac{\varphi_{3}}{2}\cos\;\varphi_{2}-\tan\;\varphi_{1}=0,\varphi_{4}=\varphi_{2},\varphi_{5}=\varphi_{1},$$

which indicates that the folding process is of one degree of freedom with one input either one of the creases *φ_i_* or the twist of facet ABCD about the *z* axis, *θ_ω_*, marked on the top view of Fig. 1*A*, ③. Alternatively, the folding and deployment of the cuboid can be controlled by the distance between facets ABCD and EFGH, which is defined as the height of the cuboid, *h*; see Fig. 1*A*, ③. The dihedral angles *φ_i_*, twist *θ_ω_*, and height *h* vs. *φ*_3_ are plotted in Fig. 1 *C*–*E*. As demonstrated in Fig. 1*A*, there are six typical configurations; that is, ① is the closed cuboid; ② is the configuration with the maximum *h*; ③ is the one with maximum dihedral angles φ_2_ (= φ_4_); ④ is an opened configuration with φ_1_ = φ_5_ = 180°, φ_2_ = φ_4_ = 90° and φ_3_ = 2arctan(1/(1 + 2μ)); ⑤ is the one with maximum dihedral angles φ_1_ = φ_5_ > 180°; and ⑥ is the flat configuration with φ_1_ = φ_5_ = 180° and φ_2_ = φ_3_ = φ_4_ = 0° as the cuboid is fully folded. Hence, as shown in Fig. 1*C*, among five creases along one limb, only φ_3_ varies monotonically from 180° to 0° during folding or vice versa during the deployment of the cuboid, while the rest of dihedral angels increase first and then decrease when the cuboid is folded into a flat configuration, so φ_3_ is taken as the input angle. Fig. 1*D* shows the relationship of *θ_ω_* vs. *φ*_3_ (*SI Appendix*, section 1), which increases monotonically with φ_3_. Yet, *h* increases from ① to ②, then decreases to zero when folded flat into ⑥ (Fig. 1*E*).

It should be noted that the curves in Fig. 1 *C*–*E* are plotted with parameters *a* = *b* for the cuboid, that is, length ratio μ = *b/a* = 1. When we take a different geometry, there is no fundamental difference in the curves of Fig. 1 *F*–*H*, where μ = 0, 0.2, 0.388, 0.417, 0.5, 0.706, 1.5, and 2 are specially selected. We can tell that a large μ corresponds to a smaller φ_3_ at ④, a large twist between ① and ④, although the total twist between ① and ⑥ is always 180°, and a large height (*h* = 2*μa*) in the deployed cuboid ① while the height of ④ is always *a*. Note that the height of ⑥ is always zero if not considering the panel thickness; thus, it is a great deployable structure with large deployable ratio along the height direction. For the cuboid with μ = 0 (*SI Appendix*, Fig. S6*A*) and 0.706, twist angle *θ_ω_* from ⑥ to ④ is 90° and 45°, respectively, which would have the potential application for electromagnetic (EM) metamaterials. When μ = 0.5, ① and ④ both have the same height as *a*.

### Realization of Multistable Structures.

No matter the selection of cuboid geometry, besides ① (φ_1_ = φ_5_ = φ_2_ = φ_4_ = 90°) and ⑥ (φ_1_ = φ_5_ = 180°, φ_2_ = φ_4_ = 0), ④ is a rather remarkable configuration with φ_1_ = φ_5_ = 180°, φ_2_ = φ_4_ = 90°, and *h* = *a*. Hence, we want to make it a stable configuration during the folding, together with ① and ⑥ to form a tristable structure.

As ① and ④ share φ_2_ = φ_4_ = 90°, we can replace creases 2 and 4 of the four limbs (eight hinges in total) with identical torsional springs of stiffness *K*_2,4_ and set the rest angle of the springs φ_20_ = φ_40_ = 90° to obtain the stable configurations ① and ④. The stored energy of each spring hinge is *U* = 1/2 *K*_2,4_ (φ_2_ − φ_20_)^2^ (*SI Appendix*, section 2*A*), leading to a total energy of *U*_2,4_ = 8*U* whose curve is plotted in Fig. 2*A* with two clear energy valleys corresponding to stable configurations ① and ④ (Movie S2). Similarly, torsional springs (stiffness *K*_1,5_) with rest angle φ_10_ = φ_50_ = 180° can also be used to replace creases 1 and 5 of the four limbs to attain the two stable configurations ④ and ⑥ corresponding to the energy valleys of curve *U*_1,5_ (Fig. 2*B* and Movie S2), because the two configurations both have φ_1_ = φ_5_ = 180°. However, if these two sets of creases 1, 5 and 2, 4 are replaced by the above-mentioned torsional springs, respectively and simultaneously, we will only obtain monostable configuration ④, because the total energy of the system *U*_1,5_ + *U*_2,4_, has only one valley, as shown in Fig. 2*C* (Movie S2), which is because the torsional springs at creases 1 and 5 are not at the 180° rest angle for configuration ①, while the torsional springs at creases 2 and 4 are also not at the 90° rest angle for configuration ⑥. Apparently, we cannot achieve a tristable structure by utilizing such simple torsional springs with one rest angle even if the ratio between *K*_2,4_ and *K*_1,5_ is tuned.

It is, however, possible to enable the cuboid to have three stable configurations, ①, ④, and ⑥, as shown in Fig. 2*D*, if the torsional springs at creases 2 and 4 are only activated between configurations ① and ④ (i.e., *U*_2,4_ > 0 and *U*_1,5_ = 0) whereas torsional springs at creases 1 and 5 are only triggered between ④ and ⑥ (i.e., *U*_2,4_ = 0 and *U*_1,5_ > 0). This can be done by creating two special elastic joints with elastic sheet materials as shown in Fig. 2*E*.

For crease 1 (and crease 5) in Fig. 2*E*, two adjacent facets, P_top_ and P_1_, are connected by a thin elastic sheet with negligible bending stiffness on one side of the facets with thickness *t*_1_. Between configurations ① and ④, there is only bending for the elastic joints 1 and 5 with φ_1_ ∈ (90°, 180°) about point 1 without any stretching, so there is no energy needed for these two joints. Between ④ and ⑥, facets P_1_ would continually rotate around axis 1 with φ_1_ > 180° as shown in Fig. 2*E*. However, the rotation is hindered by the thickness of the two adjacent panels, which leads to P_1_ rotating about axis 1′ instead of axis 1 by angle Δφ_1_ = φ_1_ − 180°. The film is therefore stretched by $\Delta x=2\sin\left(\Delta \varphi_{1}/2\right)\cdot t_{1}$, then adding to an energy consuming

[2] $$U_{\text{e}1,\text{e}5}=\frac{1}{2}K_{\text{e}1,\text{e}5}{\left(\Delta x\right)}^{2}=2K_{\text{e}1,\text{e}5}{\sin\;}^{2}\left(\Delta \varphi_{1}/2\right)\cdot t_{1}^{2}.$$

Hence, this type of elastic joint is only activated between ④ and ⑥, whose maximum energy is achieved at ⑤ where φ_1_ and φ_5_ reach maximum to generate the largest stretch on the elastic sheet at joints 1 and 5. A similar design is applied to crease 2 (and crease 4); see Fig. 2*E*. Between ④ and ⑥, φ_2_ < 90°, facet P_1_ rotates around axis 2 with respect to P_2_, which makes no stretching at all in the elastic joint. Between ① and ④, the film is stretched by $\Delta y=2\sqrt{2}\sin\;\left(\Delta \varphi_{2}/2\right)\cdot t_{2}$, as the rotation of facet P_1_ is hindered by the wedge shape of facets P_1_ and P_2_, which leads to P_1_ rotating about axis 2′ by angle Δφ_2_ = φ_2_ – 90°, then causes energy consuming

[3] $$U_{\text{e2},\text{e4}}=\frac{1}{2}K_{\text{e2},\text{e4}}{\left(\Delta y\right)}^{2}=4K_{\text{e2},\text{e4}}{\sin\;}^{2}\left(\Delta \varphi_{2}/2\right)\cdot t_{2}^{2}.$$

Hence, this elastic joint is only triggered between ① and ④ with maximum energy at ③ where φ_2_ and φ_4_ reach maximum to generate the largest stretch on the elastic sheet at joints 2 and 4.

Now we have two types of elastic joints that function in different ranges for the two sets of creases 1, 5 and 2, 4, when the kirigami cuboid moves. At the same time, we can use the same elastic joint on crease 3 by attaching to the inner sides of facets, which will never be triggered, as φ_3_ ∈ (0°, 180°) with no energy request on the joint. Based on this, a prototype made from 3-mm-thick wood panels and elastic latex film of 0.09-mm thickness demonstrates that there are indeed three stable configurations; see Fig. 2*F* and Movie S3, whose total joints’ energy *U*_e_ vs. *φ*_3_ is plotted as the red curve in Fig. 2*G* (*SI Appendix*, section 2*B*) with two energy barriers among ①, ④, and ⑥ to form a tristable structure. The energy barrier between ④ and ⑥ (at ⑤) is rather low compared with the one between ① and ④ (at ③), because Δφ_1_ = φ_1_ − 180° between ④ and ⑥ is much smaller than Δφ_2_ = φ_2_ – 90° between ① and ④, as shown in Fig. 1*C*. In order to increase the energy between ① and ④, we can increase the ratio *K*_e1,e5_/*K*_e2,e4_ as the dashed blue curve in Fig. 2*G*. The stiffness of the two types of elastic joints can be designed independently by using different sizes of the elastic sheet or different stiffnesses of the sheet material. We also can increase both energy barriers by increasing the facet thickness according to Eqs. **2** and **3**; see the black curve in Fig. 2*G*. In fact, the thickness of every facet on the cuboid can also be selected independently, which will make the cuboid have uneven outside surfaces. Note that, in the cuboid with elastic joints on the facets with thickness, the rotation axes of the hinges change from 1, 2 to 1′, 2′ when the elastic joints are active. The corresponding kinematic behaviors have been analyzed in *SI Appendix*, section 4, which is different from kinematic behaviors of the zero-thickness cuboid as we derived in Eq. **1**. However, the kinematic curves considered the facet thickness (Fig. 2 *H* and *I* and *SI Appendix*, section 4) are more or less the same as those zero-thickness ones in Fig. 1 *C* and *E* if the thicknesses *t*_1_ and *t*_2_ are small, which will vary more with the increase of thickness. It should be noted that the thickness of the top and bottom panels is not taken into consideration for the height of the structure when plotting Fig. 2*I*.

The energy barriers between states ①, ④, and *U*_S3_ at state 3, and between states ④, ⑥, and *U*_S5_ at state 5, can be programmed by length ratio μ and the thickness of the panels (Fig. 2 *J* and *K* and *SI Appendix*, section 5). Both rise with the panel thickness, while *U*_S3_ increases with μ and *U*_S5_ decreases with μ. It should be pointed out that state ②, the configuration with the maximum *h*, and state ③, the one with maximum dihedral angles φ_2_ (= φ_4_) which corresponds to the maximum energy state, do not always appear in order, as shown in Fig. 2*L*. Based on the derivation in *SI Appendix*, section 6, we can tell that state ② is between ① and ③ with μ > 0.388 and between ③ and ④ with μ < 0.388; especially, when μ = 0.388, state ② and state ③ are coincident, when the panel thickness is 5 mm.

### The Transformation among Three Stable States.

**Fig. 3.**
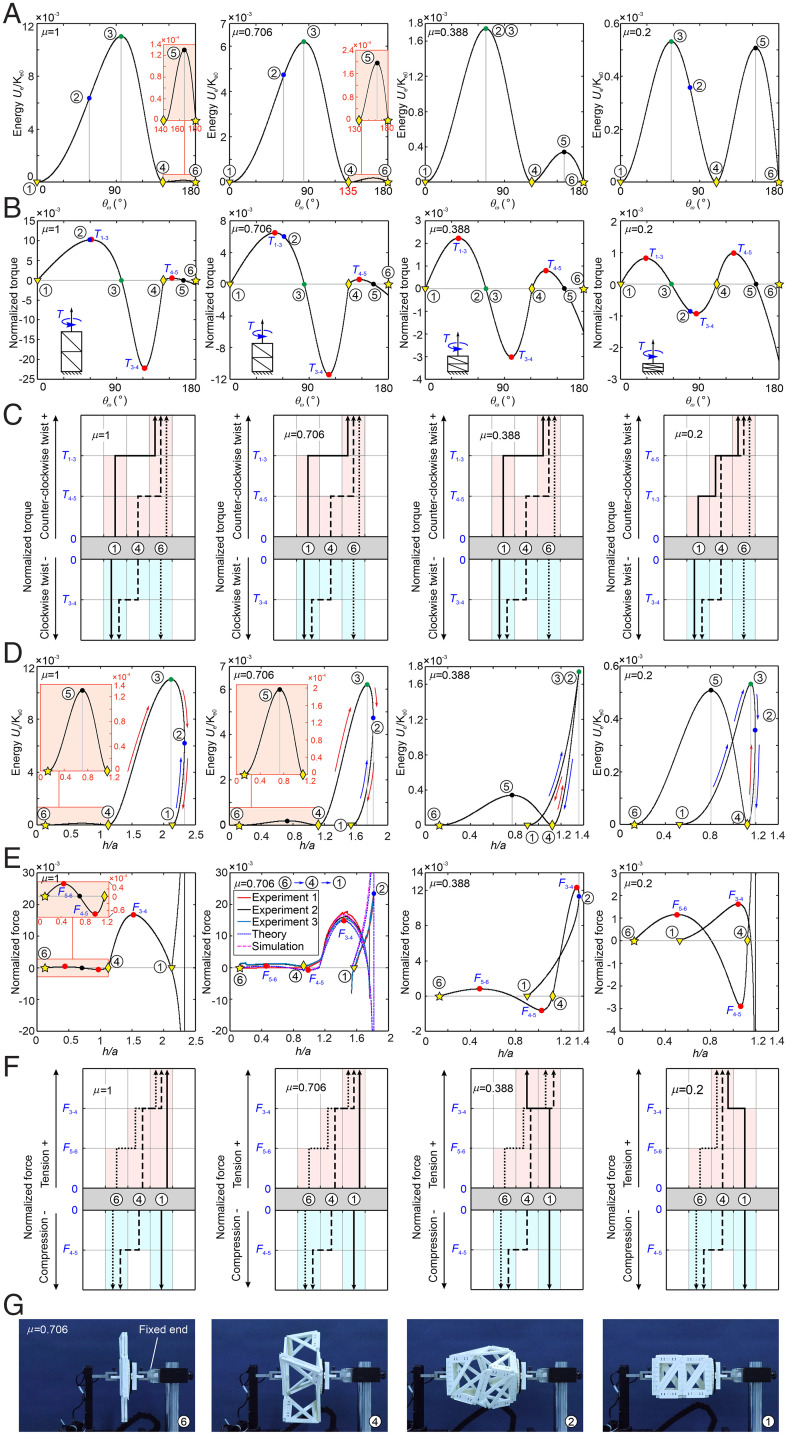
Transformation among the stable states under *z-*axial rotation or tension. (*A*) The energy vs. *θ_ω_* of the tristable structures with μ = 1, 0.706, 0.388, and 0.2. (*B*) The normalized torques vs. *θ_ω_* of the tristable structures with μ = 1, 0.706, 0.388, and 0.2, and the relationships between the stable states. (*C*) The regions of normalized torques for the transformation among three stable states (solid line starts from state ①, dashed line starts from state ④, and dotted line starts from state ⑥), where zigzag arrow lines represent the successful transformation paths, while the straight vertical lines indicate no state transformation. (*D*) The energy vs. *h/a* of the tristable structures with μ = 1, 0.706, 0.388, and 0.2, where the blue and red arrows represent the transformation between adjacent states. (*E*) The curves of normalized forces vs. *h*/*a* about the tristable structure with μ = 1, 0.706, 0.388, and 0.2. (*F*) The regions of normalized forces for the transformation among three stable states. (*G*) The experiment of structure with μ = 0.706 under tension transforming from ⑥ to ①, whose force–displacement curves are shown in *E*.

Generally, as long as the energy input to the system is larger than the energy barriers, we can easily transform it among the stable states. As analyzed above, the folding and deployment of the cuboid can be controlled by rotating φ_3_ on four limbs simultaneously; it is, in fact, not easy to realize such synchronized control because both links of φ_3_ are not fixed. Alternatively, we can twist the facet ABCD relative to EFGH about the *z* axis as *θ_ω_* increases monotonically from ① to ⑥, as demonstrated in Fig. 1 *D* and *G*. The relationship of the system energy vs. twist *θ_ω_* under different μ (*SI Appendix*, section 2*B*) is shown in Fig. 3*A*, where there are always three valleys for stable configurations ①, ④, and ⑥ and two peaks for configurations ③ and ⑤. Hence, we can fix the bottom facet and apply a counterclockwise twist with a positive torque (Fig. 3*B*) on the top facet to realize the transformation of the left-handed structure from configuration ① to ④ and then to ⑥. Here, the applied torques should overcome the energy barriers of *U*_S3_ and *U*_S5_ referring to the corresponding maximum torques *T*_1–3_ and *T*_4–5_ (Fig. 3 *B* and *C* and *SI Appendix*, section 7*A*), respectively. However, when we apply a clockwise twist on the top facet to reverse the transformation process from configuration ⑥ to ④ and then to ①, an initial tension has to also be applied at configuration ⑥, as it is a locked point with all facets coplanar for a clockwise twist noted by dotted straight lines shown in Fig. 3*C*. All the transformation paths starting from states ①, ④, and ⑥ under specific torques are noted by solid lines, dashed lines, and dotted lines respectively in Fig. 3*C* under counterclockwise or clockwise twist. Apparently, when the cuboid is right-handed, the twist and torque will be in opposite directions in order to realize the same transform among the three stable states.

Meanwhile, we can apply the tension to realize the transformation among three stable configurations, as the folding and deployment of the cuboid can be controlled by the height of the cuboid (Fig. 1 *E* and *H*) no matter its chirality. The relationship of energy vs. *h* can be obtained (*SI Appendix*, section 2*B*) and is plotted in Fig. 3*D* for μ = 1, 0.706, 0.388, and 0.2. There are obviously three valleys for stable configurations ①, ④, and ⑥ and two peaks for configurations ③ and ⑤. Therefore, we have to overcome the maximum energy at configurations ③ and ⑤ by applying the tension to input energy (Fig. 3*E* and *SI Appendix*, section 7*B*).

Yet, as discussed in Fig. 2*L*, we have to notice that the configuration ② with the maximum *h* allocates on different positions in respect to configuration ③ for different geometry μ. First, when μ > 0.388, ② is located between ① and ③. When tension is applied on the top facet (blue arrow in Fig. 3*D* with μ = 1 and 0.706) from ①, the structure moves to ②. As the energy of ② is lower than peak ③, the structure will move back to ①, instead of ④, when the force is released, so the tension force will not transform the structure from ① to ④ (solid lines in Fig. 3*F* with μ = 1 and 0.706). Conversely, when we apply tension on the top facet of ④ (red arrow in Fig. 3*D* with μ = 1 and 0.706), the structure will overcome the peak ③ and reach ②, then release this tension, and it will move to ① automatically for a stable configuration with zero energy (dashed lines in Fig. 3*F* with μ = 1 and 0.706). Second, when μ < 0.388, ② is located between ③ and ④. Tension applied on the top facet (blue arrow in Fig. 3*D* with μ = 0.2) will make the structure move from ① to ② to overcome the peak ③; then, releasing this tension, it will move to ④, for a stable configuration (solid line in Fig. 3*F* with μ = 0.2). For the reverse motion, when we apply tension on the top facet of ④ (red arrow in Fig. 3*D* with μ = 0.2), the structure will reach ② first. As the energy of ② is lower than peak ③, the structure will move back to ④, instead of to ①, so the tension force will not transform the structure from ④ to ① (dashed line in Fig. 3*F* with μ = 0.2). Third, the most interesting case happens at μ = 0.388, where φ_2_/φ_4_ (also the energy) and *h* reach the maximum at the same time; that is, configurations ② and ③ are concurrent (Fig. 3*D* with μ = 0.388). Hence, starting from ①, the tension (blue arrow in Fig. 3*D* with μ = 0.388) will drive the structure to reach ②, which is also the energy peak ③; then, releasing the tension will make it move to ④, due to dynamic inertia (solid line in Fig. 3*F* with μ = 0.388). Similarly, the tension will also do the reverse transformation (red arrow in Fig. 3*D* and dashed line in Fig. 3*F* with μ = 0.388); see Movie S3.

No matter the value of μ, the transformation between ④ and ⑥ is rather straightforward, as the height changes monotonically between these two stable configurations. Hence, the compression is applied from ④ to ⑥, and tension is applied for reverse motion.

For the structure with μ = 0.706, horizontal tensile tests (*SI Appendix*, section 8 *A* and *B*) and simulation (*SI Appendix*, section 9) with velocity 0.2 mm/s from configuration ⑥ were carried out. Fig. 3*E* with μ = 0.706 shows the force–displacement curves, which indicate the trends of the theoretical, experimental, and simulation results are consistent with three stable configurations ①, ④, and ⑥ and the limit position ②. From the folded configuration ⑥, tension is introduced to realize the transformation of three configurations from ⑥ to ① through ④ (Fig. 3*G* and Movie S4). Here, we can define two critical forces, *F*_5–6_ and *F*_3–4_, as the maximum forces requested to overcome the energy barriers of *U*_S5_ and *U*_S3_, respectively. Hence, when the tension is less than *F*_5–6_, the structure will stay at state ⑥; when the tension is between *F*_5–6_, and *F*_3–4_, the structure will be at state ④; and, when the tension is larger than *F*_3–4_, the structure will reach state ①.

### The Tessellation of Tristable Units into Metastructure with Programmable Multistable States.

By now, we have demonstrated that the transformation of three stable states on the tristable cuboid can be programmed by the geometric ratio μ, elastic joint stiffness, and thickness of panels. Regarding a unit, this cuboid can be tessellated in series to obtain a metastructure with multiple stable states.

**Fig. 4.**
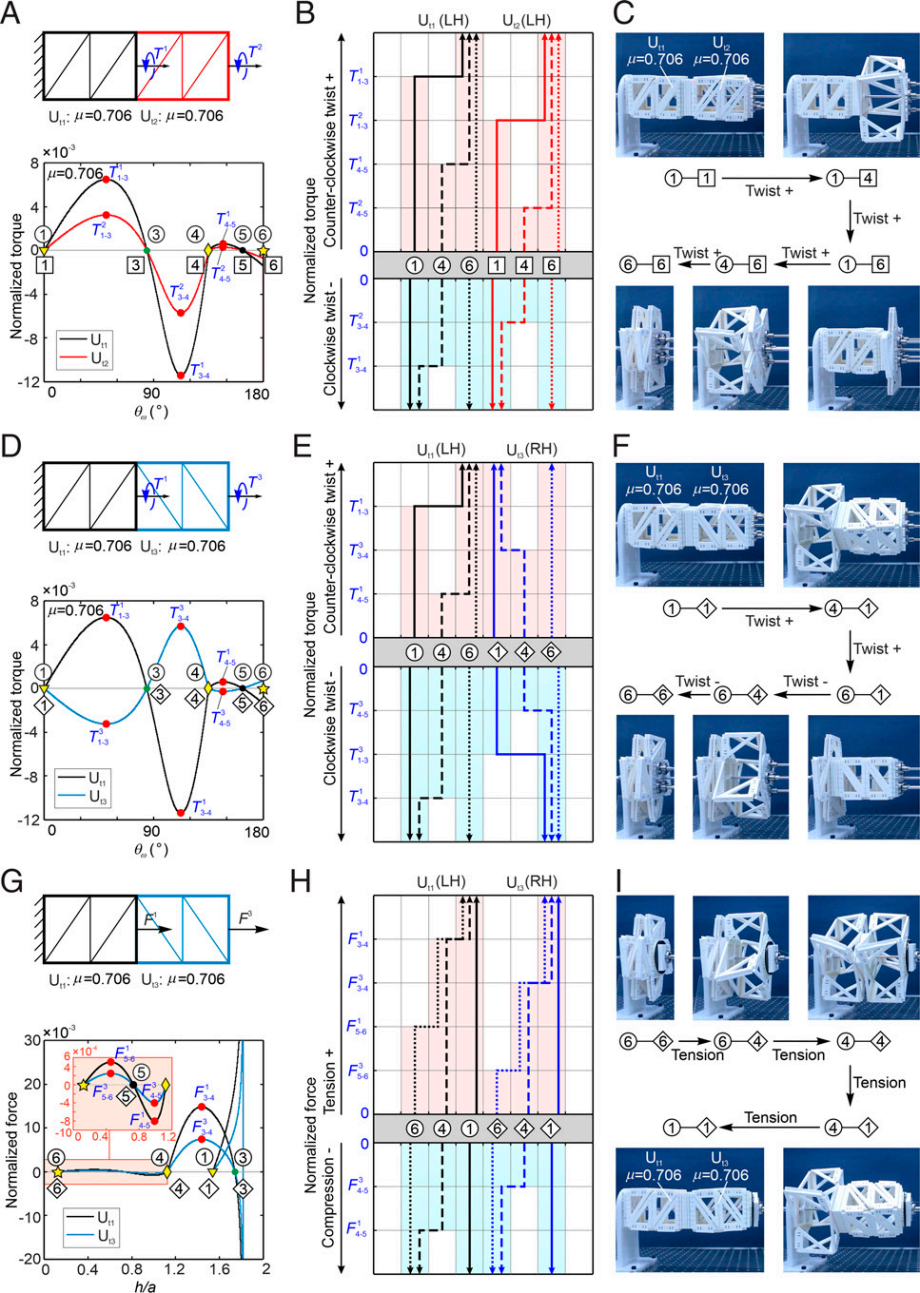
The tessellation of tristable units to program the multiple stable states controlled by input rotations or tensions. (*A*) The assembly of tristable structures left-handed U_t1_ and U_t2_ with μ = 0.706 and $K_{\text{e}1,\text{e}5}^{\text{Ut}1}\text{/}K_{\text{e}1,\text{e}5}^{\text{Ut}2}=K_{\text{e}2,\text{e}4}^{\text{Ut}1}\text{/}K_{\text{e}2,\text{e}4}^{\text{Ut}2}=2$, where the torques *T*^1^ and *T*^2^ are measured from the end of U_t1_ and U_t2_, and the corresponding curves of torques *T*^1^ or *T*^2^ vs. *θ_ω_* of the assembly. (*B*) The relationships between the stable states and the regions of normalized torques during the transformation from an arbitrary stable state in the assembly. (*C*) The experiment on the transformation of stable states from ①–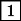 for the assembly of U_t1_ and U_t2_. (*D*–*F*) The same as in *A*–*C* but for the assembly of U_t1_ (left-handed, μ = 0.706) and U_t3_ (right-handed, μ = 0.706) with $K_{\text{e}1,\text{e}5}^{\text{Ut}1}\text{/}K_{\text{e}1,\text{e}5}^{\text{Ut}3}=K_{\text{e}2,\text{e}4}^{\text{Ut}1}\text{/}K_{\text{e}2,\text{e}4}^{\text{Ut}3}=2$ under torques. (*G*–*I*) The same as in *D*–*F* but for the same assembly under tension.

Start from a simple assembly consisting of two left-handed units U_t1_ and U_t2_, both with μ = 0.706, connected by sharing the right facet of U_t1_ and the left facet of U_t2_ (Fig. 4*A*). If the corresponding stiffness of elastic joints in units U_t1_ and U_t2_ are equal, stable states must be from ①–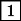 to ⑥–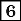 via. ④–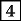, similar to a single unit. In order to obtain more stable states, the stiffness of elastic joints is set as $K_{\text{e}1,\text{e}5}^{\text{Ut}1}/K_{\text{e}1,\text{e}5}^{\text{Ut}2}=K_{\text{e}2,\text{e}4}^{\text{Ut}1}/K_{\text{e}2,\text{e}4}^{\text{Ut}2}=2$. Therefore, the corresponding transforming torque can be calculated for each unit (Fig. 4*A* and *SI Appendix*, section 7*A*), which shows that $T_{3-4}^{1}<T_{3-4}^{2}<0<T_{4-5}^{2}<T_{4-5}^{1}<T_{1-3}^{2}<T_{1-3}^{1}$ (Fig. 4*B*). Hence, as demonstrated in Fig. 4*C*, gradually applying a counterclockwise twist at the right end of this assembly at state ①–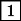 will move it to state ①-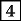 when torque is released after reaching the range between $T_{1-3}^{2}$ and $T_{1-3}^{1}$ with the twist angle between 90° and 135° (Fig. 3*A*). Next, another small torque larger than $T_{4-5}^{2}$ will make the U_t2_ transform to 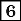, and the assembly would be in state ①–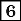. By increasing the input torque to the region larger than $T_{1-3}^{1}$, U_t1_ will reach ④, and the whole assembly will be in state ④–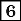 after releasing the torque when the twist angle is between 90° and 135°. Again, a further twist larger than $T_{4-5}^{1}$ will drive the assembly into state ⑥–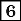 (Fig. 4*C*). The whole process shows the transformation from ①–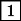 to ⑥–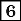 via ①–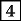, ①–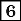, and ④–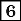 (Movie S5; the simulation is in *SI Appendix*, section 9).

When U_t2_ (left-handed, μ = 0.706) is replaced by U_t3_ (right-handed, μ = 0.706) with identical stiffness of hinges ($K_{\text{e}1,\text{e}5}^{\text{Ut}1}\text{/}K_{\text{e}1,\text{e}5}^{\text{Ut}3}=K_{\text{e}2,\text{e}4}^{\text{Ut}1}\text{/}K_{\text{e}2,\text{e}4}^{\text{Ut}3}=2$), a left-hand–right-hand (LH-RH) assembly is constructed (Fig. 4*D*). As U_t3_ is right-handed, the clockwise torque will transform it from state 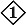 to 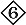 via 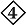. The torque–twist curve is shown in Fig. 4*D* (*SI Appendix*, section 7*A*), and the corresponding transform path is shown Fig. 4*E* for both units. When the assembly is at state ①–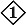, the counterclockwise twist can only activate U_t1_, and the clockwise one activates U_t3_. Thus, when counterclockwise and clockwise twists are applied to the LH-RH assembly in different sequences, we will get different transformation paths; that is, when first counterclockwise and then clockwise twists are applied successively to the LH-RH assembly, the transformation path ①–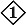, ④–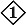, ⑥–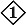, ⑥–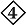, and ⑥–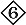 is realized, as shown in Fig. 4*F* (Movie S5; the simulation is in *SI Appendix*, section 9), while, in the opposite twist order, the transformation path is path ①–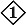, ①–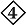, ①–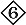, ④–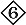, and ⑥–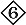 according to Fig. 4*E*, which is different from that in Fig. 4*F*. Comparing the transformation paths in Fig. 4 *C* and *F*, the two multistable structures have the same start and end stable states, while the intermediate states are totally different. Hence, the twist load sequence and the chirality of the unit can be used to program the transformation paths.

When tension and compression forces are applied to transform stable states, the chirality of the assembly will not make any difference. Hence, we take the identical assembly in Fig. 4*D* to discuss the stable-state transformation under tension/compression. Its schematic diagram and curves of forces vs. *h*/*a* are shown in Fig. 4*G* (*SI Appendix*, section 7*B*), which indicates $F_{5-6}^{3}<F_{5-6}^{1}<F_{3-4}^{3}<F_{3-4}^{1}$, and the relationships between the stable states and the ranges of tension forces for the transformations among stable states are shown in Fig. 4*H*. As the two-layer assembly cannot be twisted from stable state ⑥–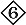 to other states due to the self-locking of panels, the transformation from stable states ⑥–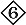 is analyzed with tension. Because of the different stiffnesses on elastic joints of U_t1_ and U_t3_, the transformation path is in the order of states ⑥–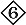, ⑥–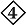, ④–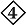, ④–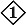, and ①–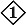 (Fig. 4*I* and Movie S6; the simulation is in *SI Appendix*, section 9). Here, by applying tension, the stable state ④–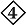 is obtained, which is not available for the torque loading mode.

**Fig. 5.**
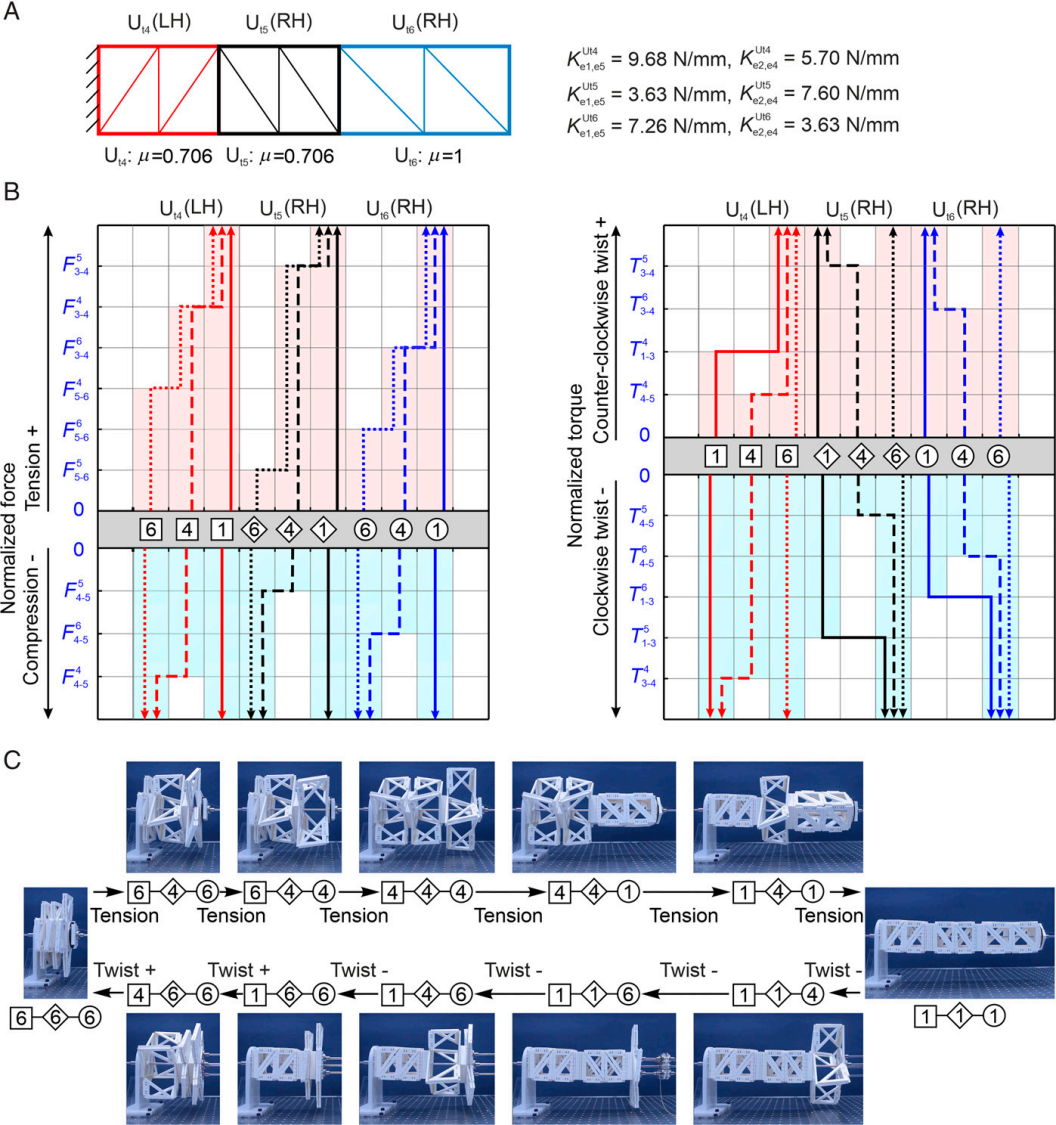
An assembly of three tristable units with multiple stable states programmed by the geometric parameters and stiffness of the elastic joints as well as loading modes. (*A*) The assembly of tristable structures left-handed U_t4_, right-handed U_t5_, both with μ = 0.706, and right-handed U_t6_ with μ = 1. (*B*) The relationships between the stable states and the regions of normalized forces/torques during the transformation from an arbitrary stable state in the assembly. (*C*) The transformation path of 12 stable states driven by tension and twists.

The examples in Fig. 4 are all taken from the identical set of design parameters in length ratio μ, elastic joint stiffness, and panel thickness to demonstrate the effect of cuboid chirality, loading modes, and sequence on the programming of stable states. It is well expected that the combination of the design parameters and loading conditions will bring in much enhanced multistable behaviors. One such example is shown in *SI Appendix*, section 10*A*. Certainly, the number of stable states increases exponentially with the number of units in the series tessellation. One such example is shown in Fig. 5, where assembly of three units with carefully selected parameters and loading modes exhibits 12 programmed stable states (*SI Appendix*, section 10*B* and Movie S7).

### The Application Demonstration Case of Tristable Metastructure for the Frequency Reconfigurable Antenna.

**Fig. 6.**
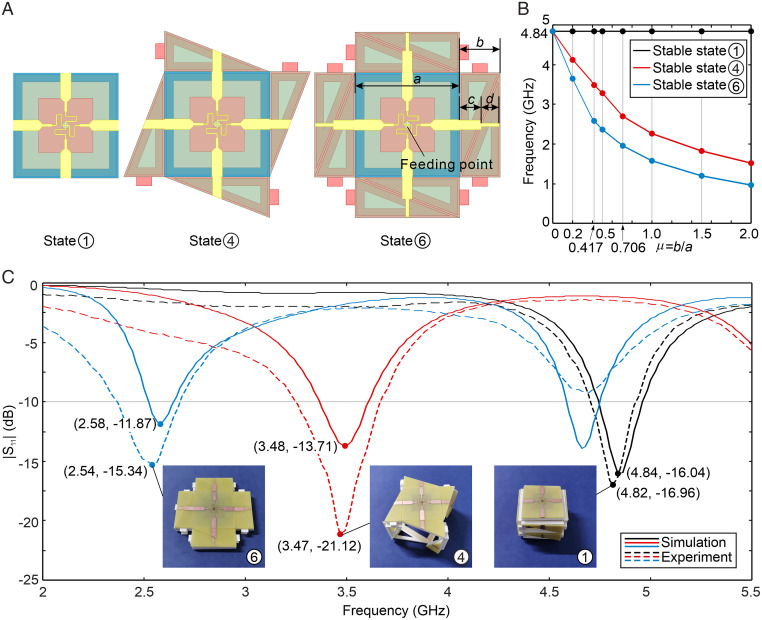
Frequency reconfigurable antenna. (*A*) The design of an antenna corresponding to three stable states with dimension parameters *a*, *b*, *c*, and *d*, in which *b*/*a* = μ, *c*/*b* = 0.541, and *d*/*b* = 0.442. (*B*) The simulation results and the measurement results of the proposed reconfigurable antenna under three stable states with corresponding dimensions of μ = 0, 0.2, 0.417, 0.5, 0.706, 1.0, 1.5, and 2.0, and *a* = 58 mm. (*C*) The experiment prototype and the simulation/measurement results of the proposed reconfigurable antenna under three stable states with corresponding dimensions of μ = 0.417 and *a* = 58 mm.

To explore the possible application of the proposed tristable metastructure, a frequency reconfigurable antenna is designed using top surfaces of a tristable kirigami cuboid with three stable states, that is, state ①, state ④, and state ⑥. The antenna is printed on the FR4 substrate with dimension parameters of *a*, *b*, *c*, and *d* under the constraint condition of *b*/*a* = μ, *c*/*b* = 0.541, and *d*/*b* = 0.442, and the antenna is fed through coaxial cable to the feeding point as shown in Fig. 6*A* (*SI Appendix*, section 11). For the fixed top square with side length *a* = 58 mm, when the antenna is configurated to state ①, the operation band of the reconfigurable antenna is centered at 4.84 GHz with reflection coefficient in decibels better than −10 dB. With the increase of μ, the corresponding antenna operation frequency decreases as the antenna length increases. For state ④ and state ⑥, the operation frequency of state ⑥ is always lower than that of state ④, as shown in Fig. 6*B*. For the 5G triple-band communication application, that is, 2.5- to 2.66-GHz, 3.3- to 3.6-GHz, and 4.8- to 5-GHz bands, we use full-wave EM simulation tools to design and achieve the optimized dimensions of μ = 0.417, that is, *a* = 58 mm, *b* = 24.2 mm, *c* = 13.1 mm, and *d* = 10.7 mm. As shown in Fig. 6*C*, for the three stable states ①, ④, and ⑥, the antenna operation frequency is centered at 4.84, 3.48, and 2.58 GHz, respectively. We did the prototype fabrication and experiments of our reconfigurable antenna. The experiment results agreed well with the simulation results, demonstrating that the proposed tristable metastructure can be used to design a high-performance reconfigurable antenna. By changing the dimensions, the antenna can be used for other triband wireless applications apart from the 5G case.

## Discussion

To conclude, we have presented a generic tristable kirigami cuboid with specially designed elastic joints, and constructed metastructures with an exponentially increased number of stable states. First, a kirigami cuboid is proposed and analyzed, to study its kinematic behaviors, which has an excellent folding/extension property, but three degrees of freedom, among which only the symmetric motion path is applicable. Second, to achieve multiple stable states, elastic joints effective in specific motion ranges have been designed by integrating the elastic sheets and kinematically switchable hinge axes when the thick panels are introduced to the kirigami cuboid. By installing such elastic joints as certain joints on the kirigami cuboid with thick facets, the resultant structure has three stable states with two energy peaks between them. Furthermore, the transformation among three stable states under uniaxial displacement and force (tension and compression) and/or twist and torque (clockwise and counterclockwise) has been discussed, and the programmability with length ratio, facet thickness, and stiffness of elastic joints has been explored. Third, by assembling tristable unit cells in series, multiple stable systems are constructed, which could be further programmed by loading mode, loading sequence, and chirality of the unit. Finally, we have demonstrated that the tristable cuboid can be applied as the base structure for a frequency reconfigurable antenna.

The kirigami cuboid with μ > 0.388 can be easily deployed and folded at the stable states, which offers an ideal deployable unit for the design of structures with large deployable ratios. The chirality in the unit cell makes it a potential candidate to design chiral structures. Meanwhile, the multistability transformation is always accompanied by a programmable stiffness, which could be applied for mechanical metamaterials (*SI Appendix*, section 12). Similar to the cuboid with a square section discussed in the paper, other kirigami prisms with polyhedral sections can be further studied to find new multistable metastructures. Moreover, their advanced engineering applications in multifunctional materials and devices, such as tunable metamaterials, deployable structures, and reconfigurable robotics, should be explored extensively.

## Materials and Methods

### The Bistable Unit.

The faces of the model in Fig. 2*A* were created from an acrylic panel (thickness is 3 mm) cut by a laser cutter (Trotec Speedy 300 25W; power: 98%; speed: 0.5%; Hz: 1,000). Hinges were 1-inch copper hinges, and the spring hinges were assembled with a torsional spring (65Mn Spring Steel; wire diameter: 1 mm; mean spring diameter: 3.5 mm; number of coils: 5) whose spring constant is supposed to be 3.21 N.mm/rad. The spring hinges’ supporters are made of acrylic panels and stick to the faces with 502 superglue.

### The Tristable Structures.

The faces of models in Fig. 2*F* were created from plywood sheets 3 mm in thickness and length *a* = 50 mm, which were cut by a laser cutter (VLS4.60 CO_2_ Laser 60W; power: 100%; speed: 10%; Hz: 500). The spring hinges were made of natural rubber latex films (thickness 0.09 mm). Those films were adhered to plywood sheets with superglue. The faces of models in Figs. 3*G* and 4 were created by acrylonitrile-butadiene-styrene from a 3D printer (Dimension Elite) with 5-mm thickness and length *a* = 80 mm. The faces are connected by polyethylene wires and Tyvek paper, and the elastic joints were made of natural rubber latex films (the per length [in millimeters] stiffness of natural rubber latex films with the thickness 0.3 mm is *K*_ep0.3_ = 0.1037N/mm^2^ from the stiffness experiment). The wires, papers, and films are adhered to sheets with LOCTTLF 401 glue. Details are provided in *SI Appendix*, section 8*A*.

## Supplementary Material

Supplementary File

Supplementary File

Supplementary File

Supplementary File

Supplementary File

Supplementary File

Supplementary File

Supplementary File

## Data Availability

All study data are included in the article and/or supporting information.
