# Supplementary material for: Kirigami-based metastructures with programmable multistability
Source: Proc Natl Acad Sci U S A. 2022 Mar 7;119(11):e2117649119. doi: 10.1073/pnas.2117649119 (PMC8931353; doi:10.1073/pnas.2117649119)
Supplement: Supplementary File [file pnas.2117649119.sapp.pdf]

PNAS
www.pnas.org

Supplementary Information for
Kirigami-based Metastructures with Programmable Multistability

Xiao Zhang[a], Jiayao Ma[a], Mengyue Li[a], Zhong You[b], Xiaoyan Wang[c], Yu Luo[c], Kaixue Ma[c], Yan Chen[a,d]
[a]School of Mechanical Engineering, Tianjin University, Tianjin, China, 300072;
[b]Department of Engineering Science, University of Oxford, Oxford, U. K., OX1 3PJ;
[c]The Tianjin Key Laboratory of Imaging and Sensing Microelectronic Technology, School of Microelectronics, Tianjin University, Tianjin 300072, China.
[d]Key Laboratory of Mechanism Theory and Equipment Design of Ministry of Education, Tianjin University, Tianjin, 300072, China

Corresponding Author: Yan Chen
Email: yan_chen@tju.edu.cn

This PDF file includes:

Supplementary text
Figures S1 to S11
Legends for Movies S1 to S7
Legends for Datasets S1 to S4
SI References

Other supplementary materials for this manuscript include the following:

Movies S1 to S7
Datasets S1 to S4

Supplementary Information Text

1. Kinematic Analysis of the Kirigami-inspired Foldable Cuboid

The kirigami is shown in Fig.S1A with four rotationally symmetrical limbs and each limb consists of five hinges. As the mechanism is a parallel mechanism, the truss method (1) is used to determine the number of mobility. For a truss without external forces and consisting of b bars and j joints, the equilibrium equation is $\boldsymbol{At} = 0$, where $\boldsymbol{A}$ is the $3j \times b$ equilibrium matrix, $\boldsymbol{t}$ is a $b \times 1$ vector of bar axial forces. If r is the rank of the matrix $\boldsymbol{A}$, the number of mobility is

$$m = 3j - 6 - r, \tag{S1}$$

The truss form of the mechanism in Fig.S1A can be derived by using bars and nodes to replace edges of facets (the black solid lines in Fig.S1A). For triangular facets, three bars will make a facet rigid and for quadrilateral facets, arbitrary points V_1 and V_2 out of the facets ABCD and EFGH are introduced. By counting, the truss form in Fig.S1A contains $j = 18$ nodes and $b = 46$ bars. When taking the configuration in system 0 (Fig.S1B), the coordinate of nodes can be obtained (Dataset S3, truss method data) and the equilibrium matrix of the mechanism can be established according to (1). Then, we obtain the rank of the matrix as $r = 45$. The number of mobility is $m = 3$.

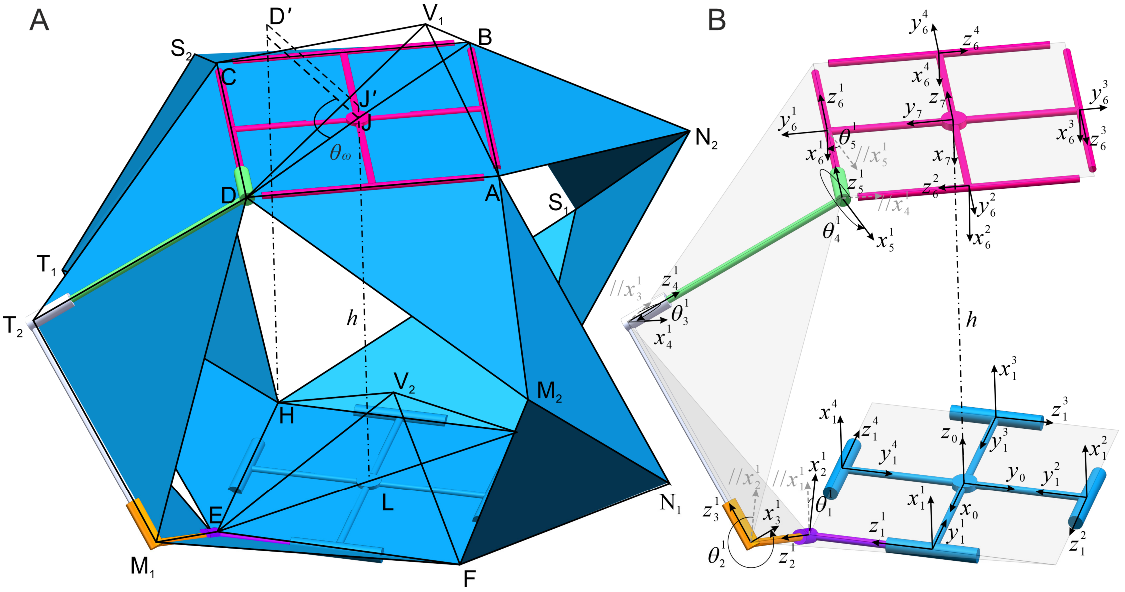

Fig. S1. The mechanism corresponding to the foldable cuboid for kinematic analysis. (A) The deployable configuration and the truss form. (B) The corresponding mechanism diagram with a limb.

As the mechanism needs three inputs to determine its configuration, it is not easy to analyze and control the motion of the mechanism directly. Here, we consider the motion with rotational symmetry in the mechanism. A deployable configuration shown in Fig.S1A is constructed where all the limbs are symmetric. Hence, their rotated angles are correspondingly equal. Here, one of the limbs with the top and bottom facets is picked up and its corresponding mechanism diagram (Fig.S1B) is also raised. Coordinate systems are

set up on this mechanism (2), as shown in Fig.S1B. Here, we chose limb 1 in Fig.S1A. The notation x_j^i, y_j^i and z_j^i are the axes of the system j on the limb i, where j=1, 2, 3, 4, 5 and i=1, 2, 3, 4. The axis z_j^i is along the axis of j th revolute joint on the limb i; the axis x_{j+1}^i is along the common normal line from z_j^i to z_{j+1}^i; the axis y_j^i can be determined by the right-hand rule; θ_j^i is the angle between x_j^i and x_{j+1}^i measured from x_j^i to x_{j+1}^i along the positive direction of z_j^i; $\alpha_{i(i+1)}$ is omitted here for simplification and they can be measured from z_j^i and z_{j+1}^i along the positive direction of x_{j+1}^i, where $\alpha_{12}=\frac{\pi}{2}$, $\alpha_{23}=\frac{3\pi}{2}$, $\alpha_{34}=\frac{\pi}{2}$, $\alpha_{34}=\frac{\pi}{2}$, $\alpha_{45}=\frac{3\pi}{2}$ and $\alpha_{56}=0$ in a limb. The mechanism with four limbs has three independent loops according to the Euler's equation

$$l=g-n+1. \tag{S2}$$

As any two limbs can form a loop, we choose the three loops, L_1L_2, L_2L_3 and L_3L_4 to carry on the further study.

For a single closed loop linkage consisting of n links, the product of the transformation matrices is equal to the 4×4 identity matrix $\boldsymbol{I}_4$, as

$$\boldsymbol{T}_{21}\boldsymbol{T}_{32}\cdots\boldsymbol{T}_{n(n-1)}\boldsymbol{T}_{1n}=\boldsymbol{I}_4. \tag{S3}$$

Hence, we can obtain the following relationships from the three loops,

$$\boldsymbol{T}_{10}^i\boldsymbol{T}_{21}^i\boldsymbol{T}_{32}^i\boldsymbol{T}_{43}^i\boldsymbol{T}_{54}^i\boldsymbol{T}_{65}^i\boldsymbol{T}_{76}^i=\boldsymbol{T}_{10}^{i+1}\boldsymbol{T}_{21}^{i+1}\boldsymbol{T}_{32}^{i+1}\boldsymbol{T}_{43}^{i+1}\boldsymbol{T}_{54}^{i+1}\boldsymbol{T}_{65}^{i+1}\boldsymbol{T}_{76}^{i+1}, \tag{S4}$$

where limb i = 1, 2, 3 and the transformation matrix $\boldsymbol{T}_{(j+1)j}^i$ about the j+1th coordinate system and the jth coordinate system are

$$\begin{aligned}
\boldsymbol{T}_{21}^i&=\boldsymbol{Tr}_z\left(0,0,\frac{a}{2}\right)\boldsymbol{R}_z\left(\theta_1^i\right)\boldsymbol{R}_x\left(\alpha_{12}^i\right),\\
\boldsymbol{T}_{32}^i&=\boldsymbol{Tr}_z\left(0,0,b\right)\boldsymbol{R}_z\left(\theta_2^i\right)\boldsymbol{R}_x\left(\alpha_{23}^i\right),\\
\boldsymbol{T}_{43}^i&=\boldsymbol{Tr}_z\left(0,0,a\right)\boldsymbol{R}_z\left(\theta_3^i\right)\boldsymbol{R}_x\left(\alpha_{34}^i\right),\\
\boldsymbol{T}_{54}^i&=\boldsymbol{Tr}_z\left(0,0,b\right)\boldsymbol{R}_z\left(\theta_4^i\right)\boldsymbol{R}_x\left(\alpha_{45}^i\right),\\
\boldsymbol{T}_{65}^i&=\boldsymbol{Tr}_z\left(0,0,\frac{a}{2}\right)\boldsymbol{R}_z\left(\theta_5^i\right)\boldsymbol{R}_x\left(\alpha_{56}^i\right),
\end{aligned} \tag{S5a}$$

and

$$
\begin{aligned}
&\boldsymbol{T}_{10}^{1}=\boldsymbol{Tr}_{x}\left(\frac{a}{2},0,0\right)\boldsymbol{R}_{x}\left(\frac{\pi}{2}\right)\boldsymbol{R}_{z}\left(\frac{\pi}{2}\right),\ \boldsymbol{T}_{67}^{1}=\boldsymbol{Tr}_{y}\left(0,\frac{a}{2},0\right),\\
&\boldsymbol{T}_{10}^{2}=\boldsymbol{Tr}_{y}\left(0,\frac{a}{2},0\right)\boldsymbol{R}_{y}\left(\frac{\pi}{2}\right)\boldsymbol{R}_{z}\left(\pi\right),\ \boldsymbol{T}_{67}^{2}=\boldsymbol{Tr}_{z}\left(0,0,-\frac{a}{2}\right)\boldsymbol{R}_{x}\left(\frac{3\pi}{2}\right),\\
&\boldsymbol{T}_{10}^{3}=\boldsymbol{Tr}_{y}\left(-\frac{a}{2},0,0\right)\boldsymbol{R}_{x}\left(\frac{3\pi}{2}\right)\boldsymbol{R}_{z}\left(\frac{3\pi}{2}\right),\ \boldsymbol{T}_{67}^{3}=\boldsymbol{Tr}_{y}\left(0,-\frac{a}{2},0\right)\boldsymbol{R}_{x}\left(\pi\right),\\
&\boldsymbol{T}_{10}^{4}=\boldsymbol{Tr}_{y}\left(0,-\frac{a}{2},0\right)\boldsymbol{R}_{y}\left(\frac{3\pi}{2}\right),\ \boldsymbol{T}_{67}^{4}=\boldsymbol{Tr}_{z}\left(0,0,\frac{a}{2}\right)\boldsymbol{R}_{x}\left(\frac{\pi}{2}\right).
\end{aligned}
\tag{S5b}
$$

Here, rotation matrices rotate vectors by an angle θ about the *x*-, *y*-, or *z*-axis of a coordinate system in homogeneous coordinates are

$$
\begin{aligned}
&\boldsymbol{R}_{x}(\theta)=\begin{bmatrix}1&0&0&0\\0&\cos\theta&-\sin\theta&0\\0&\sin\theta&\cos\theta&0\\0&0&0&1\end{bmatrix},\ \boldsymbol{R}_{y}(\theta)=\begin{bmatrix}\cos\theta&0&\sin\theta&0\\0&1&0&0\\-\sin\theta&0&\cos\theta&0\\0&0&0&1\end{bmatrix},\\
&\boldsymbol{R}_{z}(\theta)=\begin{bmatrix}\cos\theta&-\sin\theta&0&0\\\sin\theta&\cos\theta&0&0\\0&0&1&0\\0&0&0&1\end{bmatrix},
\end{aligned}
\tag{S6}
$$

and the translation matrix along vector $\boldsymbol{v}$ is

$$
\boldsymbol{Tr}_{\boldsymbol{v}}\left(v_x,v_y,v_z\right)=\begin{bmatrix}1&0&0&v_x\\0&1&0&v_y\\0&0&1&v_z\\0&0&0&1\end{bmatrix}.
\tag{S7}
$$

For the Eq. S4, we can rewrite it as

$$
\begin{aligned}
\boldsymbol{E}_{12}&=\boldsymbol{T}_{10}^{1}\boldsymbol{T}_{61}^{1}\boldsymbol{T}_{76}^{1}-\boldsymbol{T}_{10}^{2}\boldsymbol{T}_{61}^{2}\boldsymbol{T}_{76}^{2}=0,\\
\boldsymbol{E}_{23}&=\boldsymbol{T}_{10}^{2}\boldsymbol{T}_{61}^{2}\boldsymbol{T}_{76}^{2}-\boldsymbol{T}_{10}^{3}\boldsymbol{T}_{61}^{3}\boldsymbol{T}_{76}^{3}=0,\\
\boldsymbol{E}_{34}&=\boldsymbol{T}_{10}^{3}\boldsymbol{T}_{61}^{3}\boldsymbol{T}_{76}^{3}-\boldsymbol{T}_{10}^{4}\boldsymbol{T}_{61}^{4}\boldsymbol{T}_{76}^{4}=0,
\end{aligned}
\tag{S8}
$$

where $\boldsymbol{T}_{61}^{i}=\boldsymbol{T}_{21}^{i}\boldsymbol{T}_{32}^{i}\boldsymbol{T}_{43}^{i}\boldsymbol{T}_{54}^{i}\boldsymbol{T}_{65}^{i}$ with i = 1, 2, 3, 4.

The symmetric conditions of the motion indicate the corresponding rotated angles of the four limbs are equal, that is $\theta_j^1=\theta_j^2=\theta_j^3=\theta_j^4$. Therefore, we can only consider the loop L_1L_2. By introducing the symmetric condition $\theta_1^i=\theta_5^i$ and $\theta_2^i=\theta_4^i$ of the top and bottom facets and the equation $\boldsymbol{Q}_1=\boldsymbol{E}_{12}+\boldsymbol{E}_{23}$, we obtain the

$$
\begin{aligned}
&\cos^2\theta_2+\left(1+\tan^2\theta_1\right)\cos\theta_2+\sin\theta_2\tan\theta_1\left(\sin\theta_1\tan\theta_1+2\mu+\cos\theta_1\right)+\tan^2\theta_1=0,\\
&\tan\frac{\theta_3}{2}\tan\theta_1-\cos\theta_2=0,
\end{aligned}
\tag{S9}
$$

from the following elements of the matrix $\boldsymbol{Q}_1$

$$\boldsymbol{Q}_1(2,1) = -2\sin\theta_2\left(\cos\theta_1\cos\theta_2 - \sin\theta_1\sin\theta_3 + \cos\theta_1\cos\theta_2\cos\theta_3\right),$$

$$\boldsymbol{Q}_1(2,4) = -a\begin{pmatrix} 2\cos\theta_2 - \cos\theta_3 + \cos^2\theta_2 + \cos^2\theta_2\cos\theta_3 + \cos\theta_2\sin\theta_1\sin\theta_2 \\ +\cos\theta_1\sin\theta_2\sin\theta_3 + 2\mu\sin\theta_2\sin\theta_3 + \cos\theta_2\cos\theta_3\sin\theta_1\sin\theta_2 + 1 \end{pmatrix}. \quad \text{(S10)}$$

According to coordinate systems and the geometry of the model, relationships between dihedral angles and rotated angles are

$$\theta_1 = \pi - \varphi_1,\ \theta_2 = \varphi_2 - \pi,\ \theta_3 = \pi - \varphi_3. \quad \text{(S11)}$$

Correspondingly, Eq. S10 can be rewritten as

$$\begin{aligned} &\cos^2\varphi_2 - \left(1+\tan^2\varphi_1\right)\cos\varphi_2 - \sin\varphi_2\tan\varphi_1\left(\sin\varphi_1\tan\varphi_1 - 2\mu + \cos\varphi_1\right) + \tan^2\varphi_1 = 0, \\ &\tan\frac{\varphi_3}{2}\cos\varphi_2 - \tan\varphi_1 = 0. \end{aligned} \quad \text{(S12)}$$

The motion paths of the mechanism are shown in Fig. 1C and F.

According to the coordinate systems of the mechanism in Fig. S1 A and B, we can obtain the original coordinates of point D' and J' in system 0 at state ①, as

$$\mathrm{D}'_0 = \begin{pmatrix} -\frac{a}{2} & -\frac{a}{2} & 2\mu a \end{pmatrix}^T,\ \mathrm{J}'_0 = \begin{pmatrix} 0 & 0 & 2\mu a \end{pmatrix}^T. \quad \text{(S13)}$$

The homogeneous coordinate of the vector $\overrightarrow{\mathrm{J}'_0\mathrm{D}'_0}$ is

$$\overrightarrow{\mathrm{J}'_0\mathrm{D}'_0} = \begin{pmatrix} -\frac{a}{2} & -\frac{a}{2} & 0 & 1 \end{pmatrix}^T. \quad \text{(S14)}$$

Besides, we can obtain the homogeneous coordinates of points D in system 5, $\mathrm{D}_5 = \begin{pmatrix} 0 & 0 & 0 & 1 \end{pmatrix}^T$. The homogeneous coordinates of points D and J in the coordinate system 0 can be obtained with

$$\begin{aligned} \mathrm{D}_0 &= \boldsymbol{T}^1_{10}\boldsymbol{T}^1_{21}\boldsymbol{T}^1_{32}\boldsymbol{T}^1_{43}\boldsymbol{T}^1_{54}\mathrm{D}_5, \\ \mathrm{J}_0 &= \boldsymbol{T}^1_{10}\boldsymbol{T}^1_{21}\boldsymbol{T}^1_{32}\boldsymbol{T}^1_{43}\boldsymbol{T}^1_{54}\boldsymbol{T}^1_{65}\boldsymbol{T}^1_{76}\mathrm{J}_7, \end{aligned} \quad \text{(S15)}$$

where $\mathrm{J}_7 = \begin{pmatrix} 0 & 0 & 0 & 1 \end{pmatrix}^T$ is the homogeneous coordinates of point J in the coordinate system 7. According to the coordinates, the twist angle can be derived from

$$\cos\theta_\omega = \frac{\overrightarrow{\mathrm{J}_0\mathrm{D}_0}\cdot\overrightarrow{\mathrm{J}'_0\mathrm{D}'_0}}{\left|\overrightarrow{\mathrm{J}_0\mathrm{D}_0}\right|\cdot\left|\overrightarrow{\mathrm{J}'_0\mathrm{D}'_0}\right|}. \quad \text{(S16)}$$

Then, we can obtain

$$\begin{aligned} \cos\theta_\omega = &-\frac{1}{2}\cos^2\theta_2 + \frac{1}{2}\sin^2\theta_1 + \frac{1}{2}\cos^2\theta_1\cos\theta_3 + \frac{1}{2}\cos\theta_3\sin^2\theta_2 \\ &-\frac{1}{2}\cos^2\theta_2\sin^2\theta_1 - \frac{1}{2}\cos^2\theta_2\cos\theta_3\sin^2\theta_1 - \cos\theta_1\cos\theta_2\sin\theta_1\sin\theta_3 \end{aligned}. \quad \text{(S17)}$$

and its corresponding equation with dihedral angles is

$$\begin{aligned}\cos\theta_\omega = &-\frac{1}{2}\cos^2\varphi_2 + \frac{1}{2}\sin^2\varphi_1 - \frac{1}{2}\cos^2\varphi_1\cos\varphi_3 - \frac{1}{2}\cos\varphi_3\sin^2\varphi_2 \\ &-\frac{1}{2}\cos^2\varphi_2\sin^2\varphi_1 + \frac{1}{2}\cos^2\varphi_2\cos\varphi_3\sin^2\varphi_1 - \cos\varphi_1\cos\varphi_2\sin\varphi_1\sin\varphi_3\end{aligned} \quad . \quad \text{(S18)}$$

According to Eqs. S12 and S18, relationships of θ_ω *vs.* φ_3 in Fig.1D and G are derived. The z-axial coordinate of point J in the coordinate system 0 is the height of h, which is

$$h = a\mu\sin\theta_1 - a\cos\theta_1\sin\theta_2 + a\mu\left(\cos\theta_3\sin\theta_1 + \cos\theta_1\cos\theta_2\sin\theta_3\right) \quad \text{(S19)}$$

and its corresponding equation with dihedral angles is

$$h = a\mu\sin\varphi_1 - a\cos\varphi_1\sin\varphi_2 - a\mu\left(\cos\varphi_3\sin\varphi_1 - \cos\varphi_1\cos\varphi_2\sin\varphi_3\right) \quad \text{(S20)}$$

for Fig.1E and H.

2. The Stored Energy of the System

A. The stored energy of foldable cuboid with torsional springs

For a spring hinge with a torsional spring, the potential energy can be derived from

$$U = \frac{1}{2}K\left(\varphi - \varphi_0\right)^2. \quad \text{(S21)}$$

where φ_0 and φ are the rest angle and real-time angle of the spring hinge, respectively, K is the stiffness of the torsional spring which is determined by modulus of elasticity of the material E, wire diameter d_w, mean spring diameter D_m, number of active coils N and can be calculated from

$$K = \frac{\pi E d_w^4}{11520 N D_m}. \quad \text{(S22)}$$

Here, the torsional spring is manufactured by 65Mn Spring Steel with $E = 206000$ MPa, $d_w = 1$ mm, $D_m = 3.5$ mm, and $N = 5$. Its spring constant is supposed to be $K = 3.21$ N.mm/rad. For the torsional springs of stiffness $K_{2,4} = K$ for creases 2 and 4 of four limbs in the kirigami cuboid with the rest angle of the springs $\varphi_{20} = \varphi_{40} = 90°$, the stored energy of each spring hinge can be derived from

$$U = \frac{1}{2}K_{2,4}\left(\varphi_2 - \varphi_{20}\right)^2. \quad \text{(S23)}$$

The total energy of the system with eight hinges can be derived from $U_{2,4} = 8U$ whose curve is plotted in Fig.2A, where constant K_{s0} = 3.21 N.mm/rad. Similarly, for the structure only with torsional springs (stiffness $K_{1,5} = K$) on creases 1 and 5 of four limbs with rest angle, $\varphi_{10} = \varphi_{50} = 180°$, the total energy can be derived from $U_{1,5} = 8\times½\, K_{1,5}\, (\varphi_1 - \varphi_{10})^2$ with curve shown in Fig.2B. When these two sets of creases 1, 5 and 2, 4 are replaced by the above-mentioned torsional springs, respectively and simultaneously, we will only obtain monostable configuration ④ because the total energy of the system $U_{1,5} + U_{2,4}$, as shown in Fig. 2C.

B. The stored energy of thick-panel foldable cuboid with elastic joints

In Fig. 2E, each elastic joint 1 (and 5) can be stretched by $\Delta x = 2\sin\left(\Delta\varphi_1 / 2\right)\cdot t_1$ and the potential energy of each elastic joint 1(and 5) can be derived from

$$U_{\mathrm{e1,e5}} = \frac{1}{2}K_{\mathrm{e1,e5}}\left(\Delta x\right)^2 = 2K_{\mathrm{e1,e5}}\sin^2\left(\frac{\Delta\varphi_1}{2}\right)t_1^2 = 2K_{\mathrm{e1,e5}}\sin^2\left(\frac{\varphi_1}{2}-\frac{\pi}{2}\right)t_1^2. \quad \text{(S24)}$$

An elastic joint 2 (and 4) in Fig. 2E can be stretched by $\Delta y = 2\sqrt{2}\sin\left(\Delta\varphi_2 / 2\right)\cdot t_2$ and the potential energy of an elastic joint 2 (and 4) can be derived from

$$U_{\mathrm{e2,e4}} = \frac{1}{2}K_{\mathrm{e2,e4}}\left(\Delta y\right)^2 = 4K_{\mathrm{e2,e4}}\sin^2\left(\frac{\Delta\varphi_2}{2}\right)t_2^2 = 4K_{\mathrm{e2,e4}}\sin^2\left(\frac{\varphi_2}{2}-\frac{\pi}{4}\right)t_2^2. \quad \text{(S25)}$$

As there are eight elastic joints on 1(and 5) and eight elastic joints 2 (and 4), the whole stored energy of the system can be derived from

$$U_{\mathrm{e}} = 8U_{\mathrm{e1,e5}} + 8U_{\mathrm{e2,e4}} = 16K_{\mathrm{e1,e5}}\sin^2\left(\frac{\Delta\varphi_1}{2}\right)t_1^2 + 32K_{\mathrm{e2,e4}}\sin^2\left(\frac{\Delta\varphi_2}{2}\right)t_2^2. \quad \text{(S26)}$$

In the tristable structure with elastic joints in Fig. 2F, the energy equations (Eqs. 2 and 3) and twist angle equations (Eq. S34 and Eq. S41) can be derived from the results of dihedral angles from the two functional codes 'IFDoTThi1.m' in Dataset S3 and 'IFDoTThi2.m' in Dataset S3 by setting μ, a and thickness of panels. The corresponding relationships between the system energy U_{e} and angle φ_3 can be obtained from 'F2G_TriStableStr_mu1_t3_50.m' in Dataset S3, as shown in Fig. 2G.

In a tristable structure with elastic joints, the energy equations (Eqs. 2 and 3) and twist angle equations (Eq. S34 and Eq. S41) can be derived from the results of dihedral angles from the two functional codes 'IFDoTThi1.m' in Dataset S3 and 'IFDoTThi2.m' in Dataset S3 by setting μ, a and thickness of panels. The corresponding relationships between the system energy U_{e} and twist angle θ_{ω} can be derived from 'F3AD_TriStableStr_mu1_t5_ 80.m' in Dataset S3 to 'F3AD_TriStableStr_mu02_t5_80.m' in Dataset S3 under different μ = 1, 0.706, 0.388 and 0.2, as shown in Fig. 3A.

Tension along the height direction of a foldable cuboid with elastic joints can activate the transformation of stable states. Its energy equations (Eqs. 2 and 3) and height equations (Eq. S36 and Eq. S42) can obtain the results with dihedral angles from the two functional codes 'IFDoTThi1.m' in Dataset S3 and 'IFDoTThi2.m' in Dataset S3 by setting μ, a and thickness of panels. The corresponding relationships between the system energy U_{e} and height h in Fig. 3D can be derived from codes 'F3AD_TriStableStr_mu1_t5_80.m' in Dataset S3 to 'F3AD_TriStableStr_mu02_ t5_80.m' in Dataset S3 corresponding to μ = 1, 0.706, 0.388 and 0.2, respectively.

3. Tension Experiment of the Elastic Joint for Stiffness

The elastic joints with elastic sheets of the foldable cuboid are constructed in Fig. 2E. Since the sheet is very thin, its bending stiffness can be negligible. Here, we carried out an experiment of an elastic joint with an elastic sheet under tension. The specimen consists of

panels, wedges and elastic sheets with dimensions shown in Fig. S2A.

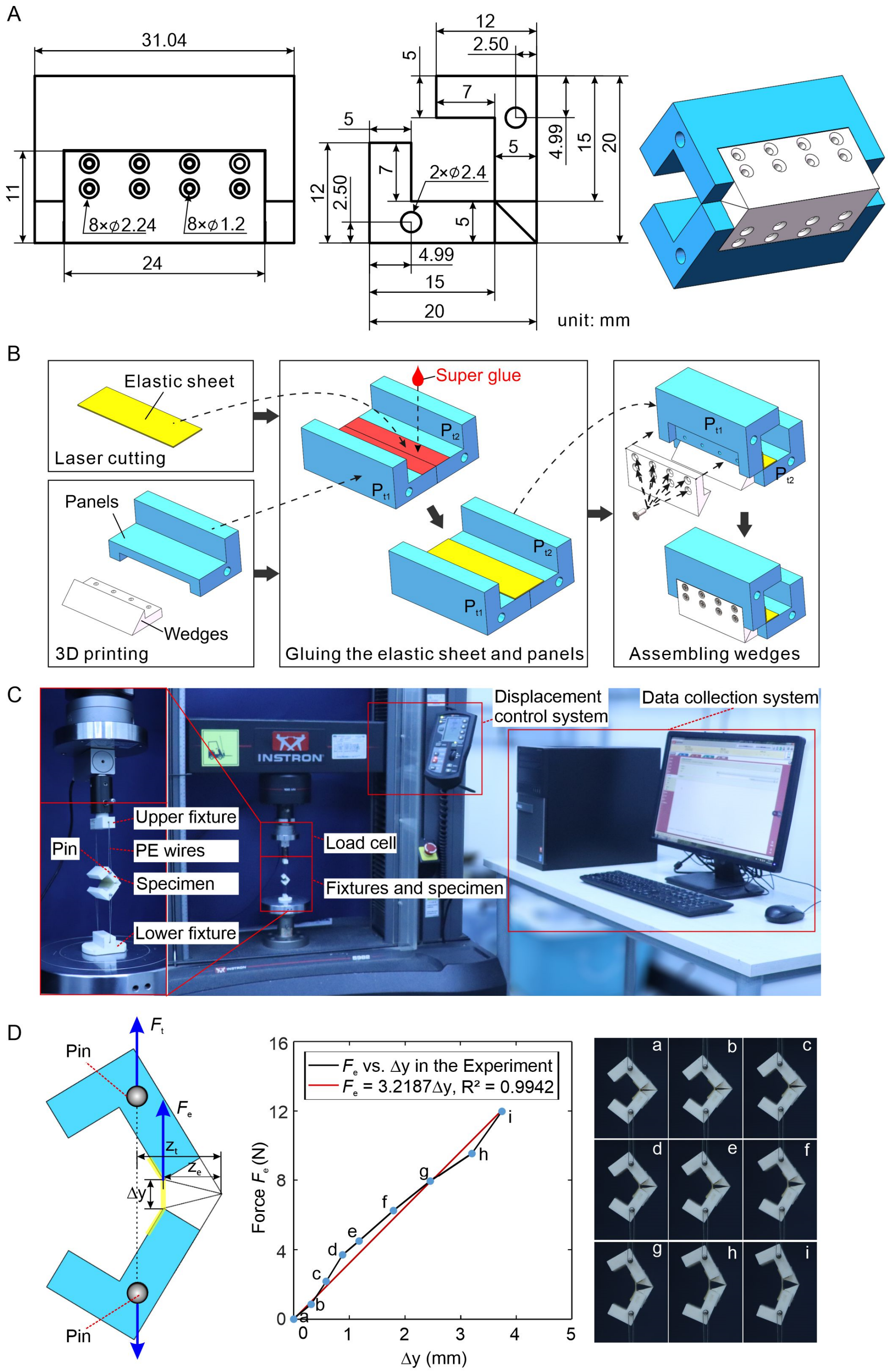

Fig. S2. Tension experiment of the elastic joint. (A) Geometric parameters of the elastic joint with latex film (thickness 0.3 mm). (B) The flowchart for the fabrication process of the elastic joint specimen. (C) The experimental setup. (D) The diagram, results and deformation configurations of the elastic joint.

The manufacturing process includes three steps, as shown in Fig. S2B. First, two panels and two wedges are fabricated by a Stratasys Dimension Elite 3D printer with ABS (acrylonitrile-butadiene-styrene), and the elastic sheet (natural rubber latex film, thickness 0.3mm) was cut by a Trotec Speedy 300 laser cutter (power: 70%, speed: 2.5%, Hz: 2000). Second, two panels P_{t1} and P_{t2} are symmetrically placed and glued together with the elastic sheet (LOCTITE 401 glue). Finally, the wedges are bolted to the panels.

The setup of the tension experiment is shown in Fig. S2C. An Instron Universal Testing Machine 5982 with a load cell of 100N is adopted for the experiment. The specimen is connected to the two fixtures on the machine through PE (polyethylene) wires. The loading speed is 1mm/min and the tension displacement is 8mm. The displacement and forces are recorded by the data collection system. The deformation process is recorded by a digital camera (Canon 70D).

Under the experiment, we can obtain the stiffness of the joint with the elastic sheet from

$$K_e = \frac{F_e}{\Delta y} = \frac{F_t z_t}{z_e \Delta y} \tag{S27}$$

whose relationships are shown in Fig. S2D (black solid polyline). This black polyline can be fitted by the red line with the equation $F_e = 3.2187\ \Delta y$ with the coefficient of determination $R^2 = 0.9942$. Hence, the stiffness of elastic joints (length 31.04 mm) with latex film (thickness $t_{e0.3} = 0.3$ mm) is 3.2187 N/mm, and the per length (mm) stiffness is $K_{ep0.3} = 0.1037$N/mm^2. As the stiffness can also be derived from $K_e = E_e t_e b_e / l_e$, where E_e is the modulus of elasticity of the material, t_e is the thickness of the film, b_e is the valid length of film on the crease, l_e is the rest width of the film in the joint, the per length stiffness of latex film with thickness $t_{e0.09}$=0.09 can be derived from $K_{ep0.09} = K_{ep0.3} t_{e0.09}/t_{e0.3} = 0.3K_{ep0.3}$. The per length stiffness of latex film with thickness $t_{e0.4}$=0.4 mm can be derived from $K_{ep0.4} = K_{ep0.3} t_{e0.4}/t_{e0.3} = 4K_{ep0.3}/3$.

4. Kinematic Analysis of Foldable Cuboid with Thick Panels

Figure. 2E has shown the changes in hinges' positions during the motion. During the motion between ① and ④, hinges 1 and 3 are kept while hinge 2 becomes the hinge 2′. When the motion is in the range between ④ and ⑥, hinge 1′ is activated and hinges 2 and 3 are kept. Similar to Fig. S1, the thick-panel structures of the two moving parts are constructed in Fig. S3 with their corresponding mechanisms of a limb for the kinematic analysis.

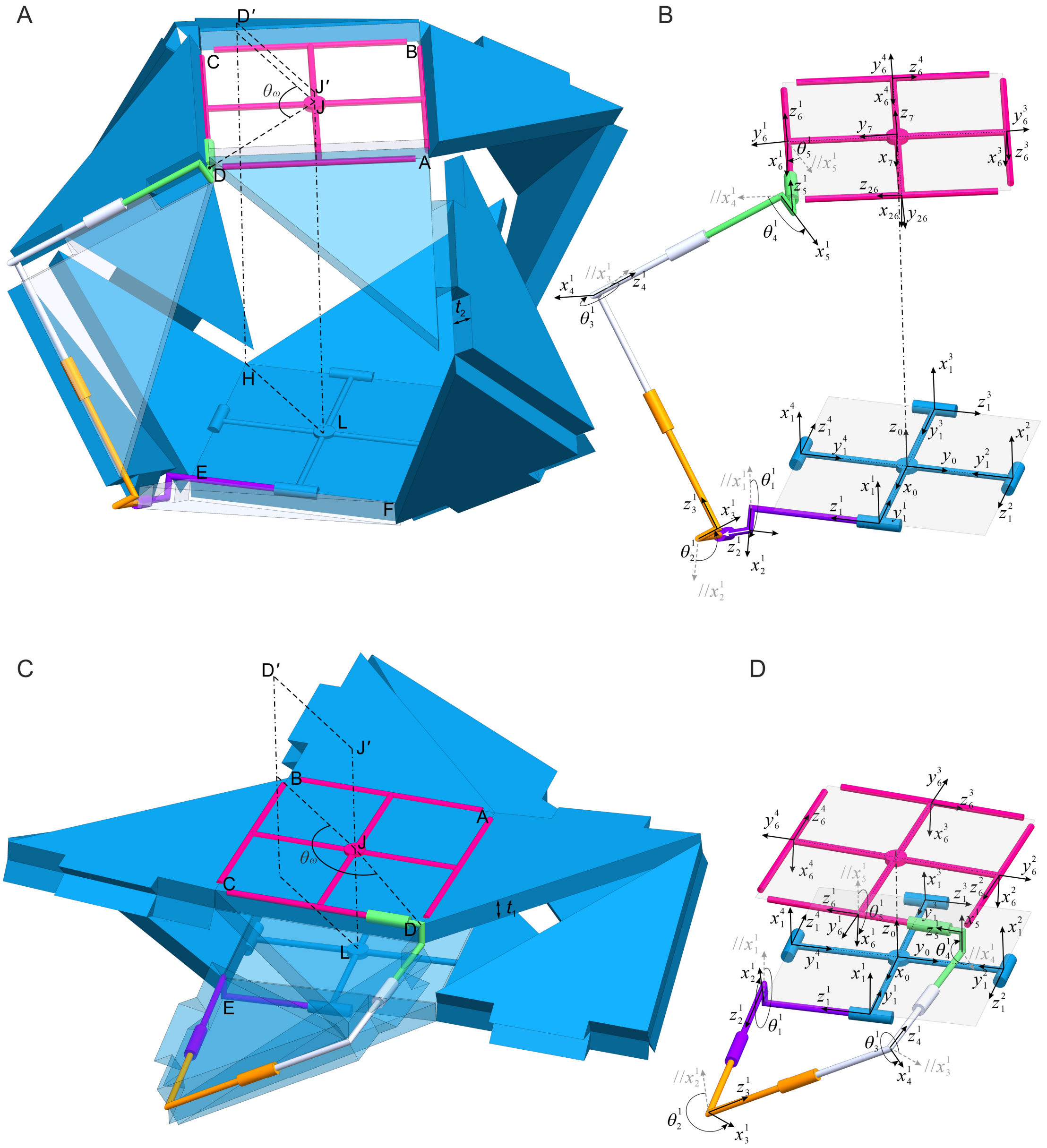

Fig. S3. The mechanism corresponding to the thick-panel structure with elastic joints corresponding to Fig. 2E. (A-B) Deployable configuration of the structure and its corresponding mechanism diagram with a limb, which is the thick-panel theoretical model for the range between ① and ④; (C-D) Deployable configuration of the structure and its corresponding mechanism diagram with a limb, which is the theoretical model for the range between ④ and ⑥.

For the motion between ① and ④, its thick-panel structure and corresponding mechanism are shown in Fig. S3A and B. Similar to Eqs.S5a and S5b, the transformation matrix $\boldsymbol{T}^{i}_{(j+1)j}$ about the j+1th coordinate system and the jth coordinate system are

$$\begin{aligned}
\boldsymbol{T}_{21}^{i} &= \boldsymbol{Tr}_z\left(0, 0, \frac{a}{2}+t_2\right)\boldsymbol{R}_z\left(\theta_1^i\right)\boldsymbol{R}_x\left(\alpha_{12}^i\right)\boldsymbol{Tr}_x\left(t_2, 0, 0\right), \\
\boldsymbol{T}_{32}^{i} &= \boldsymbol{Tr}_z\left(0, 0, b\right)\boldsymbol{R}_z\left(\theta_2^i\right)\boldsymbol{R}_x\left(\alpha_{23}^i\right)\boldsymbol{Tr}_x\left(t_2, 0, 0\right), \\
\boldsymbol{T}_{43}^{i} &= \boldsymbol{Tr}_z\left(0, 0, a+2t_2\right)\boldsymbol{R}_z\left(\theta_3^i\right)\boldsymbol{R}_x\left(\alpha_{34}^i\right)\boldsymbol{Tr}_x\left(t_2, 0, 0\right), \\
\boldsymbol{T}_{54}^{i} &= \boldsymbol{Tr}_z\left(0, 0, b\right)\boldsymbol{R}_z\left(\theta_4^i\right)\boldsymbol{R}_x\left(\alpha_{45}^i\right)\boldsymbol{Tr}_x\left(t_2, 0, 0\right), \\
\boldsymbol{T}_{65}^{i} &= \boldsymbol{Tr}_z\left(0, 0, \frac{a}{2}+t_2\right)\boldsymbol{R}_z\left(\theta_5^i\right)\boldsymbol{R}_x\left(\alpha_{56}^i\right)\boldsymbol{Tr}_x\left(t_2, 0, 0\right),
\end{aligned} \tag{S28a}$$

and

$$\begin{aligned}
&\boldsymbol{T}_{10}^{1} = \boldsymbol{Tr}_x\left(\frac{a}{2}, 0, 0\right)\boldsymbol{R}_x\left(\frac{\pi}{2}\right)\boldsymbol{R}_z\left(\frac{\pi}{2}\right),\ \boldsymbol{T}_{67}^{1} = \boldsymbol{Tr}_y\left(0, \frac{a}{2}, 0\right), \\
&\boldsymbol{T}_{10}^{2} = \boldsymbol{Tr}_y\left(0, \frac{a}{2}, 0\right)\boldsymbol{R}_y\left(\frac{\pi}{2}\right)\boldsymbol{R}_z\left(\pi\right),\ \boldsymbol{T}_{67}^{2} = \boldsymbol{Tr}_z\left(0, 0, -\frac{a}{2}\right)\boldsymbol{R}_x\left(\frac{3\pi}{2}\right), \\
&\boldsymbol{T}_{10}^{3} = \boldsymbol{Tr}_y\left(-\frac{a}{2}, 0, 0\right)\boldsymbol{R}_x\left(\frac{3\pi}{2}\right)\boldsymbol{R}_z\left(\frac{3\pi}{2}\right),\ \boldsymbol{T}_{67}^{3} = \boldsymbol{Tr}_y\left(0, -\frac{a}{2}, 0\right)\boldsymbol{R}_x\left(\pi\right), \\
&\boldsymbol{T}_{10}^{4} = \boldsymbol{Tr}_y\left(0, -\frac{a}{2}, 0\right)\boldsymbol{R}_y\left(\frac{3\pi}{2}\right),\ \boldsymbol{T}_{67}^{4} = \boldsymbol{Tr}_z\left(0, 0, \frac{a}{2}\right)\boldsymbol{R}_x\left(\frac{\pi}{2}\right).
\end{aligned} \tag{S28b}$$

Considering the symmetric condition $\theta_1^i + \pi = \theta_5^i$ and $\theta_2^i = \theta_4^i$ according to the systems and substituting Eqs.S28a and S28b into Eq.S4, according to $\boldsymbol{Q}_1 = \boldsymbol{E}_{12} + \boldsymbol{E}_{23}$, we obtain

$$\begin{aligned}
&\cos\theta_2\left(a\cos\theta_2 - a + 2t_2\sin\theta_2 - 2t_2 + 2t_2\cos\theta_2\right) + a\left(\cos\theta_1 - 2\mu\right)\sin\theta_2\tan\theta_1 \\
&+\left(a + 2t_2 - \left(a+2t_2\right)\cos\theta_2 + \left(a\sin\theta_1 - 2t_2\right)\sin\theta_2\right)\tan^2\theta_1 = 0, \\
&\tan\frac{\theta_3}{2}\cos\theta_2 = \tan\theta_1.
\end{aligned} \tag{S29}$$

from the following elements of the matrix $\boldsymbol{Q}_1$

$$\begin{aligned}
&\boldsymbol{Q}_1\left(2,1\right) = -2\sin\theta_2\left(-\cos\theta_1\cos\theta_2 + \sin\theta_1\sin\theta_3 + \cos\theta_1\cos\theta_2\cos\theta_3\right), \\
&\boldsymbol{Q}_1(2,4) = -a\begin{pmatrix} 2\cos\theta_2 + \cos\theta_3 + \cos^2\theta_2 - \cos^2\theta_2\cos\theta_3 - \cos\theta_2\sin\theta_1\sin\theta_2 \\ -\cos\theta_1\sin\theta_2\sin\theta_3 + 2\mu\sin\theta_2\sin\theta_3 + \cos\theta_2\cos\theta_3\sin\theta_1\sin\theta_2 + 1 \end{pmatrix} \\
&-2t_2\begin{pmatrix} \cos^2\theta_2 + \sin\theta_2\cos\theta_2 + 2\cos\theta_2 - \cos\theta_3 + \sin\theta_2 + \cos\theta_3\sin\theta_2 \\ +\cos^2\theta_2\cos\theta_3 + \cos\theta_2\cos\theta_3\sin\theta_2 \end{pmatrix}.
\end{aligned} \tag{S30}$$

According to the relationships between dihedral angles and rotated angles

$$\theta_1 = -\varphi_1,\ \theta_2 = \varphi_2,\ \theta_3 = -\varphi_3, \tag{S31}$$

Eq. S29 can be rewritten as

$$\begin{aligned}&\cos\varphi_2\left(a\cos\varphi_2-a+2t_2\sin\varphi_2-2t_2+2t_2\cos\varphi_2\right)+a\left(-\cos\varphi_1+2\mu\right)\sin\varphi_2\tan\varphi_1\\&+\left(a+2t_2-\left(a+2t_2\right)\cos\varphi_2-\left(a\sin\varphi_1+2t_2\right)\sin\varphi_2\right)\tan^2\varphi_1=0,\\&\tan\frac{\varphi_3}{2}\cos\varphi_2=\tan\varphi_1.\end{aligned}\tag{S32}$$

For the twist angle of the structure, we can derive it from Eqs. S15 and S16 based on the homogeneous coordinates of points D in system 5, $D_5=\begin{pmatrix}0 & 0 & t & 1\end{pmatrix}^T$, as

$$\begin{aligned}\cos\theta_\omega=&-\frac{1}{2}\cos^2\theta_2+\frac{1}{2}\sin^2\theta_1-\frac{1}{2}\cos^2\theta_1\cos\theta_3-\frac{1}{2}\cos\theta_3\sin^2\theta_2\\&-\frac{1}{2}\cos^2\theta_2\sin^2\theta_1+\frac{1}{2}\cos^2\theta_2\cos\theta_3\sin^2\theta_1-\cos\theta_1\cos\theta_2\sin\theta_1\sin\theta_3\end{aligned}\tag{S33}$$

and its corresponding equation with dihedral angles is

$$\begin{aligned}\cos\theta_\omega=&-\frac{1}{2}\cos^2\varphi_2+\frac{1}{2}\sin^2\varphi_1-\frac{1}{2}\cos^2\varphi_1\cos\varphi_3-\frac{1}{2}\cos\varphi_3\sin^2\varphi_2\\&-\frac{1}{2}\cos^2\varphi_2\sin^2\varphi_1+\frac{1}{2}\cos^2\varphi_2\cos\varphi_3\sin^2\varphi_1-\cos\varphi_1\cos\varphi_2\sin\varphi_1\sin\varphi_3\end{aligned}.\tag{S34}$$

For the height h, it should be calculated by the z-axial coordinate of point J in the coordinate system 0 and plus the thickness of the top and bottom panels, as

$$\begin{aligned}h=&t_2\cos\theta_1-a\mu\sin\theta_1+t_2\cos\theta_2\left(\sin\theta_1\sin\theta_3+\cos\theta_1\cos\theta_2\cos\theta_3\right)\\&-t_2\sin\theta_2\left(\sin\theta_1\sin\theta_3-\cos\theta_1\cos\theta_2+\cos\theta_1\cos\theta_2\cos\theta_3\right)+t_2\cos\theta_1\cos\theta_2\\&+t_2\sin\theta_1\sin\theta_3+au\left(\cos\theta_3\sin\theta_1-\cos\theta_1\cos\theta_2\sin\theta_3\right)\\&-\left(a+2t_2\right)\cos\theta_1\sin\theta_2+t_2\cos\theta_1\sin^2\theta_2+t_2\cos\theta_1\cos\theta_2\cos\theta_3+2t_2.\end{aligned}\tag{S35}$$

Its corresponding equation with dihedral angles is

$$\begin{aligned}h=&t_2\cos\varphi_1+a\mu\sin\varphi_1+t_2\cos\varphi_2\left(\sin\theta_1\sin\theta_3+\cos\varphi_1\cos\varphi_2\cos\varphi_3\right)\\&-t_2\sin\varphi_2\left(\sin\varphi_1\sin\varphi_3-\cos\varphi_1\cos\varphi_2+\cos\varphi_1\cos\varphi_2\cos\varphi_3\right)+t_2\cos\varphi_1\cos\varphi_2\\&+t_2\sin\varphi_1\sin\varphi_3-a\mu\left(\cos\varphi_3\sin\varphi_1-\cos\varphi_1\cos\varphi_2\sin\varphi_3\right)\\&-\left(a+2t_2\right)\cos\varphi_1\sin\varphi_2+t_2\cos\varphi_1\sin^2\varphi_2+t_2\cos\varphi_1\cos\varphi_2\cos\varphi_3+2t_2\end{aligned}\tag{S36}$$

For the motion between ④ and ⑥, its thick-panel structure and corresponding mechanism are shown in Fig. S3C and D. The transformation matrices are

$$\begin{aligned}
&\boldsymbol{T}_{21}^{i}=\boldsymbol{Tr}_{z}\left(0,0,\frac{a}{2}\right)\boldsymbol{R}_{z}\left(\theta_{1}^{i}\right)\boldsymbol{R}_{x}\left(\alpha_{12}^{i}\right)\boldsymbol{Tr}_{x}\left(t_{1},0,0\right),\\
&\boldsymbol{T}_{32}^{i}=\boldsymbol{Tr}_{z}\left(0,0,b\right)\boldsymbol{R}_{z}\left(\theta_{2}^{i}\right)\boldsymbol{R}_{x}\left(\alpha_{23}^{i}\right),\\
&\boldsymbol{T}_{43}^{i}=\boldsymbol{Tr}_{z}\left(0,0,a\right)\boldsymbol{R}_{z}\left(\theta_{3}^{i}\right)\boldsymbol{R}_{x}\left(\alpha_{34}^{i}\right),\\
&\boldsymbol{T}_{54}^{i}=\boldsymbol{Tr}_{z}\left(0,0,b\right)\boldsymbol{R}_{z}\left(\theta_{4}^{i}\right)\boldsymbol{R}_{x}\left(\alpha_{45}^{i}\right)\boldsymbol{Tr}_{x}\left(t_{1},0,0\right),\\
&\boldsymbol{T}_{65}^{i}=\boldsymbol{Tr}_{z}\left(0,0,\frac{a}{2}\right)\boldsymbol{R}_{z}\left(\theta_{5}^{i}\right)\boldsymbol{R}_{x}\left(\alpha_{56}^{i}\right),
\end{aligned}\tag{S37a}$$

and

$$\begin{aligned}
&\boldsymbol{T}_{10}^{1}=\boldsymbol{Tr}_{x}\left(\frac{a}{2},0,0\right)\boldsymbol{R}_{x}\left(\frac{\pi}{2}\right)\boldsymbol{R}_{z}\left(\frac{\pi}{2}\right),\ \boldsymbol{T}_{67}^{1}=\boldsymbol{Tr}_{y}\left(0,\frac{a}{2},0\right),\\
&\boldsymbol{T}_{10}^{2}=\boldsymbol{Tr}_{y}\left(0,\frac{a}{2},0\right)\boldsymbol{R}_{y}\left(\frac{\pi}{2}\right)\boldsymbol{R}_{z}\left(\pi\right),\ \boldsymbol{T}_{67}^{2}=\boldsymbol{Tr}_{z}\left(0,0,-\frac{a}{2}\right)\boldsymbol{R}_{x}\left(\frac{3\pi}{2}\right),\\
&\boldsymbol{T}_{10}^{3}=\boldsymbol{Tr}_{y}\left(-\frac{a}{2},0,0\right)\boldsymbol{R}_{x}\left(\frac{3\pi}{2}\right)\boldsymbol{R}_{z}\left(\frac{3\pi}{2}\right),\ \boldsymbol{T}_{67}^{3}=\boldsymbol{Tr}_{y}\left(0,-\frac{a}{2},0\right)\boldsymbol{R}_{x}\left(\pi\right),\\
&\boldsymbol{T}_{10}^{4}=\boldsymbol{Tr}_{y}\left(0,-\frac{a}{2},0\right)\boldsymbol{R}_{y}\left(\frac{3\pi}{2}\right),\ \boldsymbol{T}_{67}^{4}=\boldsymbol{Tr}_{z}\left(0,0,\frac{a}{2}\right)\boldsymbol{R}_{x}\left(\frac{\pi}{2}\right),
\end{aligned}\tag{S37b}$$

where $\alpha_{12}=\frac{\pi}{2}$, $\alpha_{23}=\frac{3\pi}{2}$, $\alpha_{34}=\frac{3\pi}{2}$, $\alpha_{34}=\frac{3\pi}{2}$, $\alpha_{45}=\frac{\pi}{2}$, $\alpha_{56}=0$ and $\theta_4^i=\theta_2^i$, $\theta_5^i=\pi+\theta_1^i$. According to the following elements of the matrix $\boldsymbol{Q}_1$

$$\begin{aligned}
&\boldsymbol{Q}_1\left(2,1\right)=-2\sin\theta_2\left(\cos\theta_1\cos\theta_2+\sin\theta_1\sin\theta_3-\cos\theta_1\cos\theta_2\cos\theta_3\right),\\
&\boldsymbol{Q}_1(2,4)=-a\begin{pmatrix}2\cos\theta_2+\cos\theta_3+\cos^2\theta_2+\cos^2\theta_2\cos\theta_3-\cos\theta_2\sin\theta_1\sin\theta_2\\ +\cos\theta_1\sin\theta_2\sin\theta_3-2\mu\sin\theta_2\sin\theta_3+\cos\theta_2\cos\theta_3\sin\theta_1\sin\theta_2+1\end{pmatrix}\\
&+t_1\sin2\theta_2-2t_1\cos\theta_2\cos\theta_3\sin\theta_2,
\end{aligned}\tag{S38}$$

the relationships between kinematic variables can be derived as

$$\begin{aligned}
&\left(-2t_1\sin\theta_2+a+a\cos\theta_2+a\sin\theta_1\sin\theta_2\right)\tan^2\theta_1+a\cos\theta_2+a\cos^2\theta_2\\
&+a\sin\theta_2\tan\theta_1\left(\cos\theta_1+2\mu\right),\\
&\tan\frac{\theta_3}{2}\cos\theta_2=-\tan\theta_1.
\end{aligned}\tag{S39}$$

Its corresponding equations with dihedral angles are

$$\begin{aligned}
&\left(2t_1\sin\varphi_2+a-a\cos\varphi_2+a\sin\varphi_1\sin\varphi_2\right)\tan^2\varphi_1-a\cos\varphi_2+a\cos^2\varphi_2\\
&-a\sin\varphi_2\tan\varphi_1\left(-\cos\varphi_1+2\mu\right),\\
&\tan\frac{\varphi_3}{2}\cos\varphi_2=-\tan\varphi_1,
\end{aligned}\tag{S40}$$

according to $\theta_1=\pi-\varphi_1,\ \theta_2=\varphi_2-\pi,\ \theta_3=-\varphi_3$.

Similar to the Eqs. S34 and S36, the following equations are derived. For the twist angle based on $D_5=(0\ 0\ 0\ 1)^T$, we obtain

$$\begin{aligned}\cos\theta_\omega &= \frac{1}{2}\sin^2\varphi_1-\frac{1}{2}\cos^2\varphi_2-\frac{1}{2}\cos^2\varphi_1\cos\varphi_3-\frac{1}{2}\cos\varphi_3\sin^2\varphi_2 \\ &-\frac{1}{2}\cos^2\varphi_2\sin^2\varphi_1+\frac{1}{2}\cos^2\varphi_2\cos\varphi_3\sin^2\varphi_1-\cos\varphi_1\cos\varphi_2\sin\varphi_1\sin\varphi_3.\end{aligned} \tag{S41}$$

For height h based on point J, we get

$$\begin{aligned}h &= a\mu\sin\varphi_1-t_1\cos\varphi_2\left(\sin\varphi_1\sin\varphi_3+\cos\varphi_1\cos\varphi_2\cos\varphi_3\right)-t_1\cos\varphi_1-a\cos\varphi_1\sin\varphi_2 \\ &-a\mu\left(\cos\varphi_3\sin\varphi_1-\cos\varphi_1\cos\varphi_2\sin\varphi_3\right)-t_1\cos\varphi_1\sin^2\varphi_2+2t_1.\end{aligned} \tag{S42}$$

According to the Eqs. S29, S31, S33, function codes about the relationships of geometric and kinematic variables are constructed, named 'IFDoTThi2.m' in Dataset S3 for motion between state ① to state ④ considering the motion range of the angles. The relationships of geometric and kinematic variables can be derived, when the geometric parameters, length ratio μ, length a and thickness t_2. Similarly, codes named 'IFDoTThi1.m' in Dataset S3 for motion between state ④ to state ⑥ are constructed from the Eqs. S39. Combining the motion results from the two function codes, curves of Fig. 2H and I can be derived from 'F2HI_TristableAnalysis_vsph3_t0t6.m' in Dataset S3.

5. Analysis of Energy Barriers of Tristable Structures

According to Eqs. S24 to S26, the energy barriers of the system between states ① and ④, U_{S3} at state 3, and between states ④ and ⑥, U_{S5} at state 5, can be derived from

$$U_{S3}=32K_{e2,e4}\sin^2\left(\frac{\Delta\varphi_{2,3}}{2}\right)t_2^2=16K_{e2,e4}\left(1-\cos\left(\varphi_{2,3}-\frac{\pi}{2}\right)\right)t_2^2=16K_{e2,e4}\left(1-\sin\varphi_{2,3}\right)t_2^2 \tag{S43}$$

and

$$U_{S5}=16K_{e1,e5}\sin^2\left(\frac{\Delta\varphi_{1,5}}{2}\right)t_1^2=16K_{e1,e5}\sin^2\left(\frac{\varphi_{1,5}}{2}-\frac{\pi}{2}\right)t_1^2=8K_{e1,e5}\left(1+\cos\varphi_{1,5}\right)t_1^2. \tag{S44}$$

Here, $\Delta\varphi_{2,3}$ and $\varphi_{2,3}$ are angles $\Delta\varphi_2$ and φ_2 (Fig. 2E) in state ③, $\Delta\varphi_{1,5}$ and $\varphi_{1,5}$ are angles $\Delta\varphi_1$ and φ_1 (Fig. 2E) in state ⑤. From Eq. S32, we can obtain the angle $\varphi_{2,3}$ is only determined by t_2 and μ in state ③, when the section length a is defined, i.e., U_{S3} can be programmed by length ratio μ and thickness of panels t_2 without considering the stiffness of elastic joints. As the relationships of angles are implicit functions (Eqs. S32 and S40), $\varphi_{2,3}$ is the maximum φ_2 which can be derived from the kinematic analysis of the foldable cuboid under MATLAB function 'max', the energy barrier U_{S3} can be derived from the code 'F2J_EnergyUs3VSmu_thickness_80.m' in Dataset S3 under varying length ratio μ

and thickness of panels t_2, as shown in Fig. 2J. Similarly, from Eq. S40, we can obtain the angle $\varphi_{1,5}$ is only determined by t_1 and μ in stable state ⑤, when the section length a is defined, i.e., U_{S5} can be programmed by length ratio μ and thickness of panels t_1 without considering the stiffness of elastic joints. Similarly, the energy barrier U_{S5} can be derived from the code 'F2K_EnergyUs5VSmu_thickness_80.m' in Dataset S3 under varying length ratio μ and thickness of panels t_1, as shown in Fig. 2K. Here, all the elastic joints are supposed to be with latex film (thickness 0.3 mm), the length $a = 80$ mm, and the real length of the elastic sheets in a joint is supposed to be $a_0 = 70$ mm, and the stiffness is supposed to be $K_{\mathrm{e2,e4}}=\mu K_{\mathrm{e1,e5}}=\mu K_{\mathrm{ep0.3}}a_0$.

6. The Relationships among Relative Position of States, Length Ratio and Thickness

Figures 1C and F show φ_3 gradually increases from 0° to 180°during the deployed sequence from state ⑥ to state ①, which indicates an arbitrary state of the folded cuboid corresponds to a unique value of angle φ_3. Therefore, the angle φ_3 can be used to represent the relative positions of the critical states ① to ⑥. As the state ②, the configuration with the maximum h, and state ③, the one with maximum dihedral angles φ_2 (= φ_4) which corresponds to the maximum energy state, are in the range between state ① and ④, and do not always appear in order, angles φ_3's in the state ② and state ③ are chosen to analyze their relative positions under the variation of μ and thickness of panels t_2. The relationships among φ_{32}, φ_{33}, μ and t_2 are derived from the codes 'F2L_muVSpositionOFstate2and3_3D.m' in Dataset S3 with 'IFDoTThi2.m' in Dataset S3, as shown in Fig. 2L and Fig. S4A-B. From the relationships among φ_3 in state ② (the blue surface), φ_3 in state ③ (the black surface), and t_2 in Fig. S4 A-B, we find the intersection of the two surfaces approaches a straight line which shows the influence of thickness on the relative position of two stable states ② and ③ can be negligible. Then, a foldable cuboid with the panel thickness 5 mm is chosen to show the relative position of state ② and state ③, the state ② is between ① and ③ with $\mu > 0.388$, between ③ and ④ with $\mu < 0.388$, and coincident with ③ with $\mu = 0.388$, as shown in Fig. S4C ('FS4C_muVSpositionOFstate2and3.m' in Dataset S3).

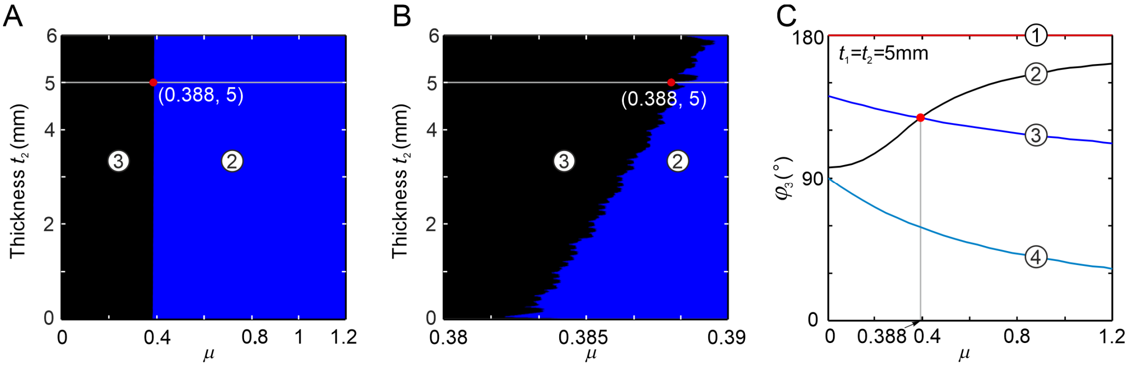

Fig. S4. The relationships among the relative position of states ② and ③, length ratio and thickness. (A) The relative position of states ② and ③ under the variation of length ratio and thickness from the negative *z*-axis view of Fig. 2L. (B) The variation of the relative position in the range μ from 0.38 to 0.39. (C) The angles φ_3's of states ①, ②, ③, ④ under different μ, which indicates the state ② is between ① and ③ with $\mu > 0.388$, between ③ and ④ with $\mu < 0.388$, and coincident with ③ with $\mu = 0.388$, when the panel thickness is 5 mm.

7. Torques and Forces of Thick-Panel Tristable Structures with Elastic Joints

A. Torques *vs.* twist θ_ω of the thick-panel tristable structures

In a tristable structure with elastic joints, the torques of a unit can be obtained by calculating the derivatives of U_e to θ_ω based on Eq. S34 and Eq. S41, as

$$T = \begin{cases} T_{e2,e4} = \dfrac{dU_{e2,e4}}{d\theta_\omega} = \dfrac{dU_{e2,e4} / d\varphi_2}{d\theta_\omega / d\varphi_2}, & \text{between states ① and ④} \\ T_{e1,e5} = \dfrac{dU_{e1,e5}}{d\theta_\omega} = \dfrac{dU_{e1,e5} / d\varphi_1}{d\theta_\omega / d\varphi_1}, & \text{between states ④ and ⑥} \end{cases} . \tag{S45}$$

For the assembly between states ① and ④, the derivative of energy $U_{e2,e4}$ to φ_2 can be derived from Eq. S26, as

$$\frac{dU_{e2,e4}}{d\varphi_2} = -16K_{e2,e4}t_2^2 \cos\varphi_2 . \tag{S46}$$

From Eqs. S32 and S34, we find the θ_ω is the function of dihedral angles φ_1, φ_2 and φ_3, while φ_1 is the function of dihedral angles φ_2 and φ_3 is the function of dihedral angles φ_1, φ_2. With the derivative of φ_1 to φ_2 in Eq. S32, we obtained

$$\frac{d\varphi_1}{d\varphi_2}=-\frac{\cos\varphi_1\begin{pmatrix}-\cos\varphi_2\sin\varphi_1\left(a-2a\mu\cos\varphi_1+2t_2\sin\varphi_1\right)+\left(a+2t_2\right)\sin\varphi_2\\+\cos^2\varphi_1\left(2t_2\cos\left(2\varphi_2\right)-\left(a+2t_2\right)\sin\left(2\varphi_2\right)\right)\end{pmatrix}}{\begin{pmatrix}a\left(-1+2\mu\cos\varphi_1\right)\sin\varphi_2-a\sin^2\varphi_1\sin\varphi_2\\-2\sin\varphi_1\left(-a-2t_2+\left(a+2t_2\right)\cos\varphi_2+2t_2\sin\varphi_2\right)\end{pmatrix}}.\text{(S47)}$$

According to the derivative of φ_3 to φ_2 in Eq. S32 with Eq. S47, we obtained

$$\frac{d\varphi_3}{d\varphi_2}=\frac{2\cos^2\left(\frac{\varphi_3}{2}\right)\begin{pmatrix}2t_2\cos\left(2\varphi_2\right)+\left(a+2t_2\right)\sec^2\varphi_1\sin\varphi_2-\left(a+2t_2\right)\sin\left(2\varphi_2\right)\\+2a\mu\cos\varphi_2\tan\varphi_1-a\cos\varphi_2\sec\varphi_1\tan\varphi_1-2t_2\cos\varphi_2\tan^2\varphi_1\end{pmatrix}}{\begin{pmatrix}\cos\varphi_2\begin{pmatrix}-4\left(a+2t_2\right)\sin^2\left(\frac{\varphi_2}{2}\right)\tan\varphi_1\\+\sin\varphi_2\left(-2a\mu+a\sec\varphi_1+4t_2\tan\varphi_1+a\sin\varphi_1\tan\varphi_1\right)\end{pmatrix}\\+\sin\varphi_2\tan\left(\frac{\varphi_3}{2}\right)\end{pmatrix}}.\text{(S48)}$$

Then, from the derivative of θ_ω to φ_2 in Eq. S34, we obtained

$$\frac{d\theta_\omega}{d\varphi_2}=-\frac{1}{2\sin\theta_\omega}\begin{pmatrix}2\frac{d\varphi_1}{d\varphi_2}\cos\varphi_1\sin\varphi_1+2\frac{d\varphi_1}{d\varphi_2}\cos\varphi_1\cos\varphi_3\sin\varphi_1\\-2\frac{d\varphi_3}{d\varphi_2}\cos\varphi_1\cos\varphi_2\cos\varphi_3\sin\varphi_1-2\cos\varphi_2\cos\varphi_3\sin\varphi_2\\-2\cos\varphi_2\left(-1+\left(-1+\cos\varphi_3\right)\sin^2\varphi_1\right)\sin\varphi_2+\frac{d\varphi_3}{d\varphi_2}\cos^2\varphi_1\sin\varphi_3\\-2\frac{d\varphi_1}{d\varphi_2}\cos^2\varphi_1\cos\varphi_2\sin\varphi_3+2\frac{d\varphi_1}{d\varphi_2}\cos\varphi_2\sin^2\varphi_1\sin\varphi_3\\+2\cos\varphi_1\sin\varphi_1\sin\varphi_2\sin\varphi_3+\frac{d\varphi_3}{d\varphi_2}\sin^2\varphi_2\sin\varphi_3\\+\cos^2\varphi_2\left(2\frac{d\varphi_1}{d\varphi_2}\cos\varphi_1\left(-1+\cos\varphi_3\right)\sin\varphi_1-\frac{d\varphi_3}{d\varphi_2}\sin^2\varphi_1\sin\varphi_3\right)\end{pmatrix}.\text{(S49)}$$

Substituting Eqs. S47 and S48 to Eq. S49, and then combining Eqs. S46 and S49 according to Eq. 45, we can obtain the torque equation between states ① and ④ in the codes 'F3BE_DerivationsForForceTorqueEq_StableStates14.nb' in Dataset S3. Similarly, the torque equation between states ④ and ⑥ can be obtained from codes 'F3BE_DerivationsForForceTorqueEq_StableStates46.nb' in Dataset S3. According to the torque equations, torques T *vs.* θ_ω with different μ can be derived from Eq. S45, as shown in Fig. 3B (codes 'F3_TorqueK_mu1_t5_80.m' in Dataset S3 to 'F3_TorqueK_mu02_t5_80.m' in Dataset S3 corresponding to μ = 1, 0.706, 0.388 and 0.2, respectively). In addition, the curves in Fig. 4A and D are derived from 'F4A_TorqueK_mu0706_0706_LHU1_LHU2_t5_80.m' and 'F4D_TorqueK_mu0706_

0706_LHU1_RHU3_t5_80.m', respectively. Here, critical torques for the transformations between stable states are noted by T_{1-3}, T_{3-4}, T_{4-5}. Normalized torques are derived from $T/\mathrm{K_{t0}}$, where $\mathrm{K_{t0}} = K_{\mathrm{ep0.3}}a^3 = 0.1037\times80^3 = 53094$ N.mm.

B. Forces *vs.* height *h* of the thick-panel tristable structures

Tension along the height direction of a foldable cuboid with elastic joints can activate the transformation of stable states. The tension forces can be derived from the derivation of the U_e to h based on Eq. S36 and Eq. S42, as

$$F=\begin{cases} F_{\mathrm{e2,e4}}=\dfrac{dU_{\mathrm{e2,e4}}}{dh}=\dfrac{dU_{\mathrm{e2,e4}}/d\varphi_2}{dh/d\varphi_2}, & \text{between states ① and ④} \\ F_{\mathrm{e1,e5}}=\dfrac{dU_{\mathrm{e1,e5}}}{dh}=\dfrac{dU_{\mathrm{e1,e5}}/d\varphi_1}{dh/d\varphi_1}, & \text{between states ④ and ⑥} \end{cases}. \tag{S50}$$

The derivative of h to φ_2 in Eq. S36 is

$$\frac{dh}{d\varphi_2}=\left(\begin{aligned} &\sin\varphi_1\left(\frac{d\varphi_3}{d\varphi_2}t_2\cos\varphi_3\left(1+\cos\varphi_2-\sin\varphi_2\right)+a\mu\frac{d\varphi_3}{d\varphi_2}\sin\varphi_3+t_2\left(-\cos\varphi_2-\sin\varphi_2\right)\sin\varphi_3\right) \\ &+\frac{d\varphi_1}{d\varphi_2}\cos\varphi_1\left(a\mu-a\mu\cos\varphi_3+t_2\left(1+\cos\varphi_2-\sin\varphi_2\right)\sin\varphi_3\right) \\ &-\frac{d\varphi_1}{d\varphi_2}\sin\varphi_1\left(\begin{aligned}&t_2+t_2\cos^2\varphi_2\cos\varphi_3-\left(a+2t_2\right)\sin\varphi_2+t_2\sin^2\varphi_2 \\ &+\cos\varphi_2\left(t_2+t_2\sin\varphi_2+\cos\varphi_3\left(t_2-t_2\sin\varphi_2\right)+a\mu\sin\varphi_3\right)\end{aligned}\right) \\ &+\cos\varphi_1\left(\begin{aligned}&-\left(a+2t\right)\cos\varphi_2+2t_2\cos\varphi_2\sin\varphi_2 \\ &-2t_2\cos\varphi_2\cos\varphi_3\sin\varphi_2 \\ &-t_2\frac{d\varphi_3}{d\varphi_2}\cos^2\varphi_2\sin\varphi_3-\sin\varphi_2\left(\begin{aligned}&t_2+t_2\sin\varphi_2+\cos\varphi_3\left(t_2-t_2\sin\varphi_2\right) \\ &+a\mu\sin\varphi_3\end{aligned}\right) \\ &+\cos\varphi_2\left(\begin{aligned}&t_2\cos\varphi_2+a\mu\frac{d\varphi_3}{d\varphi_2}\cos\varphi_3-t_2\cos\varphi_2\cos\varphi_3 \\ &-\frac{d\varphi_3}{d\varphi_2}\left(t_2-t_2\sin\varphi_2\right)\sin\varphi_3\end{aligned}\right)\end{aligned}\right)\end{aligned}\right). \tag{S51}$$

Substituting Eqs. S47 and S48 to Eq. S51, and then combining Eqs. S46 and S51, the force equation between states ① and ④ can be obtained according to Eq. S50 with the codes 'F3BE_DerivationsForForceTorqueEq_StableStates14.nb'. Similarly, the force equation between states ④ and ⑥ can be obtained from the codes 'F3BE_DerivationsForForceTorqueEq_StableStates46.nb'. According to the force equations, forces *vs.* h with different μ can be derived based on Eq. S50, as shown in Fig. 3E from the codes 'F3_ForceK_mu1_t5_80.m' in Dataset S3 to

‘F3_ForceK_mu02_t5_80.m’ in Dataset S3 corresponding to μ = 1, 0.706, 0.388 and 0.2, respectively. In addition, the curves in Fig. 4G are derived from ‘F4G_ForceK_mu0706_ 0706_LHU1_RHU3_t5_80.m’. Here, critical forces for the transformations between stable states are noted by F_{5-6}, F_{4-5}, F_{3-4}. Normalized forces are derived from F/K_{f0}, where constant $K_{f0} = K_{ep0.3}a^2 = 0.1037\times80^2 = 663.68$ N.

8. Experiment for Thick-Panel Tristable Structure and Their Assemblies

A. Fabrication of the foldable cuboids and tristable structures

Based on the geometric parameters in Fig. S5A, the panels of the specimen are fabricated by a Stratasys Dimension Elite 3D printer with ABS, whereas the elastic sheets are cut by a Trotec Speedy 300 laser cutter. Then the specimen is assembled following the process in Fig. S5B. First, one P_{top} panel is connected to four P_1 panels through four elastic sheets and PE wires for hinges 1 or 5 with LOCTITE 401 glue. Second, four P_2 panels are respectively connected to the four P_1 panels through four elastic sheets for hinges 2 and 4. Third, four P_2 and four P_3 panels are respectively joined by Tyvek paper. Fourth, the subassembly of P_1, P_2, P_3, P_{top} panels, and that of P_{bot} and P_4 panels are assembled with elastic sheets for hinges 2 and 4. Finally, sixteen wedges are installed at hinges 2 and 4.

The tristable wood structure in Fig. 2F is assembled similar to Fig. S5B with natural rubber latex film adhesive to plywood sheets. The assemblies of two units in Fig. 4 are assembled the same as that in Fig. S5B. For the assemblies of U_{t1}&U_{t2} (Fig. 4C) and U_{t1}&U_{t3} (Fig. 4F and I), the elastic joints are made of latex films (thickness 0.3mm), where the latex films for U_{t2} and U_{t3} are cut with holes (Fig. S5A) to obtain $K_{e1,e5}^{Ut1}/K_{e1,e5}^{Ut2}=K_{e2,e4}^{Ut1}/K_{e2,e4}^{Ut2}=2$ and $K_{e1,e5}^{Ut1}/K_{e1,e5}^{Ut3}=K_{e2,e4}^{Ut1}/K_{e2,e4}^{Ut3}=2$, respectively.

As the parameters μ = 0, 0.2, 0.388, 0.417, 0.5, 0.706, 1, 1.5, 2 are specially selected, there is no fundamental difference in the curves of Fig. 1F-H. For the ideal case with $\mu = 0$ ($b = 0$, $a \neq 0$), axes of hinges 1, 2 and 3 on each limb will intersect at a point, and axes of hinges 3, 4 and 5 on each limb will intersect at another point and there is physical interference of the corner joints at states ① and ⑥. Hence, the SolidWorks® model is constructed, as shown in Fig. S6A. When $\mu = 2$ a prototype with a = 80 mm was fabricated according to the process in Fig. S5B, its stable states are shown in Fig. S6B. Here, a prototype of a smaller scale tristable unit with a = 8mm and $\mu = 1$ was fabricated according to the process in Fig. S5B, in which the panels of the tristable unit are made from carbon fiber board (thickness 0.2mm) and the elastic joints are made from Tyvek paper (thickness 0.142mm). Three stable states of this unit are shown in Fig. S6C.

B. Uniaxial tension experiment for a tristable structure

For the tristable structure with a = 80mm and $\mu = 0.706$, a specimen is fabricated (Fig. S5B) and a uniaxial tension experiment is carried out. A horizontal testing machine shown in Fig. S7A is utilized to avoid the influence of gravity. The load cell is 50N with an accuracy of 0.5%. Two fixtures are used to connect the specimen to the machine, where

the left one is fixed to the load cell and the right one has a rotational degree of freedom about the *z*-axis (Fig. 1A) of the specimen. A two-step loading process is adopted, one is tensioning the specimen by 136.72 mm from stable state ⑥ to the max height state ② and the other is compressing the specimen by 24.4 mm from state ② to states ①. The loading rate is selected to be 0.2mm/s. The deformation process is recorded by a digital camera (Canon 70D).

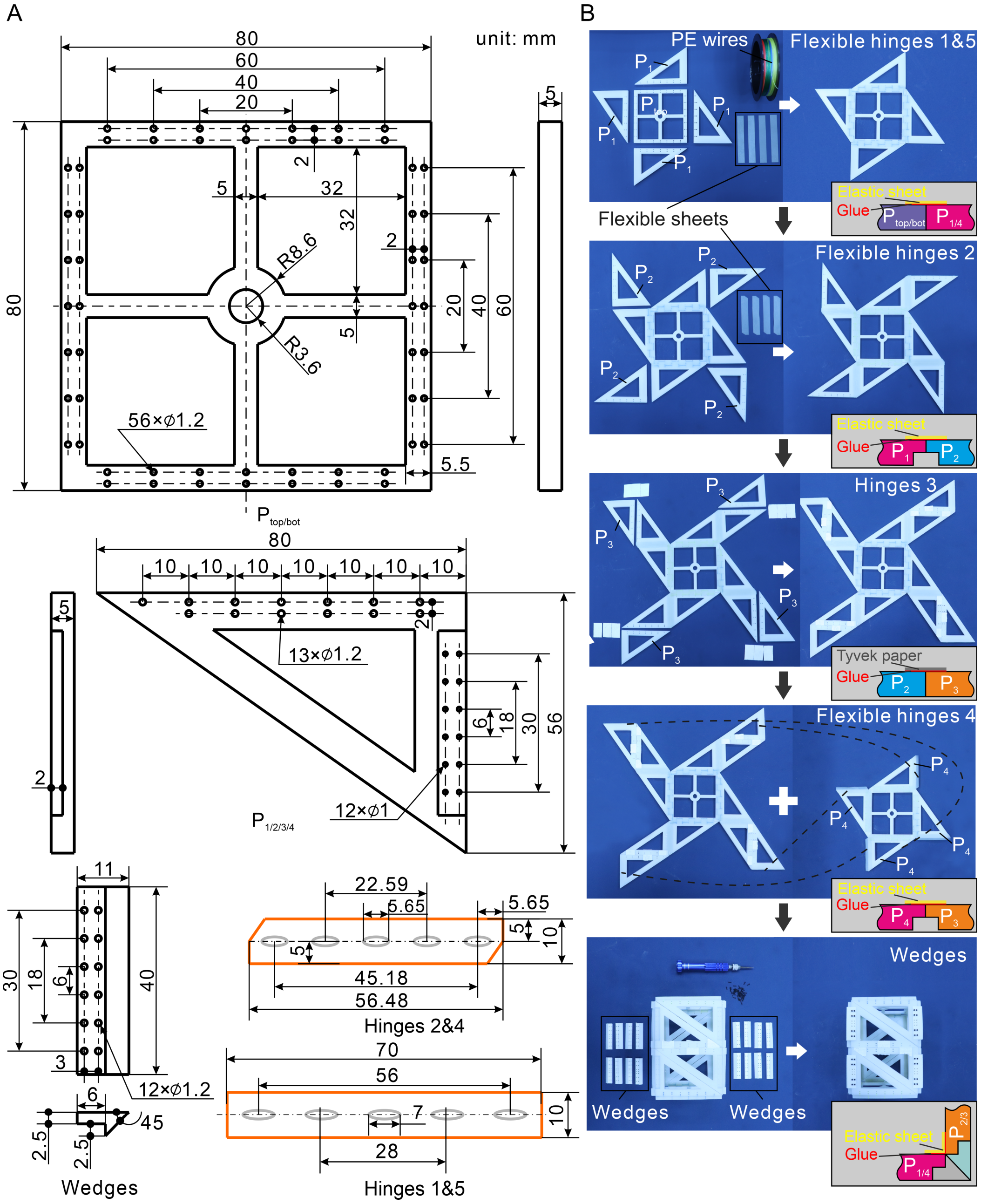

Fig. S5. The design and fabrication of a tristable structure with μ = 0.706 and a = 80 mm. (A) The dimensions of the panels, elastic sheets, and wedges. (B) The fabrication process.

C. Experiment for the assemblies in Fig. 4

Units of the assembly with a = 80mm and μ = 0.706 are fabricated according to Fig. S5B. The setup of the experiment on a horizontal testing machine is shown in Fig. S7B. The fixed supporter is used to fix the left end of U_{t1} and the right supporter is used to support the specimen through a metal shaft to avoid the influence of gravity. For the tension experiment, the load cell was connected to the right end of U_{t3} through a rotational fixture by a PE wire (diameter 0.181 mm). The load cell is 50N with an accuracy of 0.5%. The loading rate is 0.2mm/s. For the tension process, displacement of 220 mm was set up and the assembly realise the transformation from ⑥-◇6 to ④-◇2 via ⑥-◇4 and ④-◇4, and overcome the state ◇3 of U_{t3}. Then, for the release process, displacement of -40 mm was setup, the assembly went to state ④-◇1. By tensioning 80 mm, U_{t1} overcame the state ③, and the assembly got to state ①-◇1 by releasing displacement of -40 mm. The displacement and forces were recorded by the data collection system. The deformation process was recorded by a digital camera (Canon 70D). For the twisting process, the setup is similar to that in Fig. S7B, where the rotation is applied to the right end of the assembly through a cylinder with aramid wires and metal shafts connected to the assembly (Movie S5).

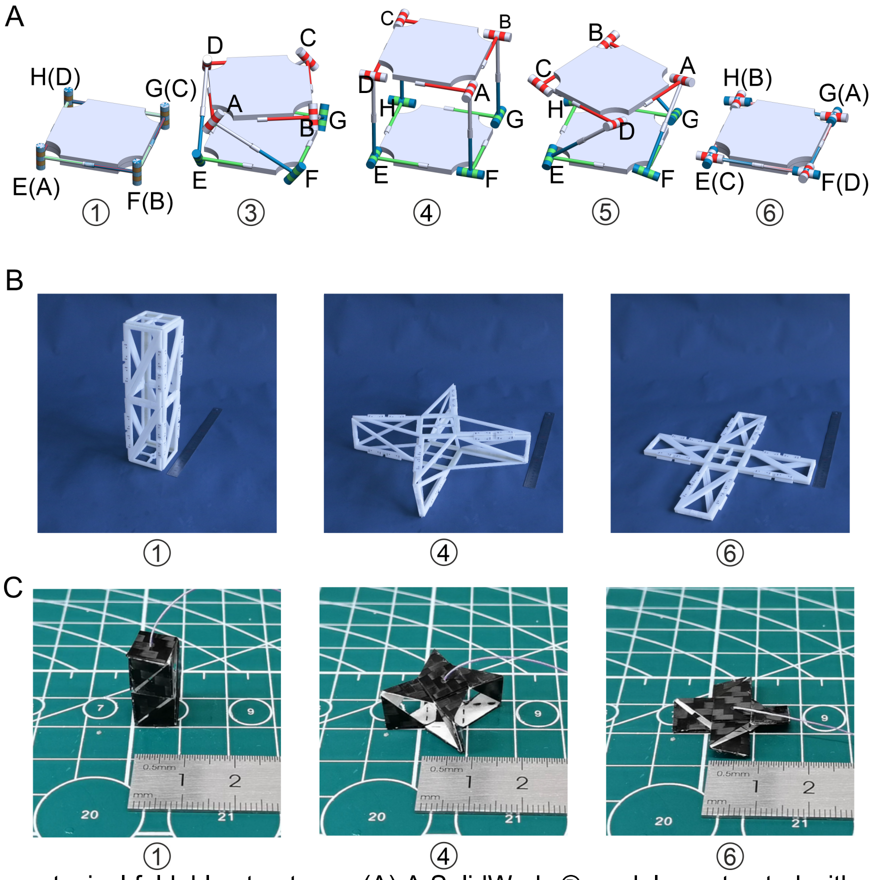

Fig. S6. Three typical foldable structures. (A) A SolidWorks® model constructed with μ = 0. (B) A prototype with a = 80 mm, μ = 2. (C) A prototype with a = 8 mm, μ = 1.

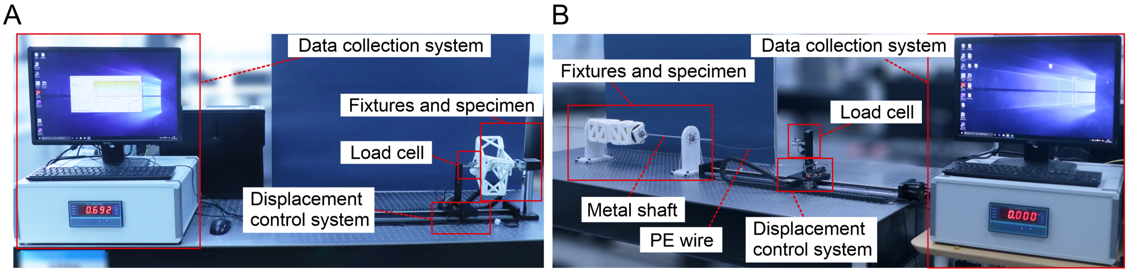

Fig. S7. The setup of the tension experiment. (A) The setup of the uniaxial tension experiment. (B) The setup of the uniaxial tension experiment for assemblies of tristable units.

9. Simulation for the Tristable Structures and Their Assemblies

SolidWorks® (Dassault Systèmes S.A, France) (3) is used to simulate the single unit and the assemblies. For a single unit, the panels are modeled as rigid bodies. Joints 1, 2, 4 and 5 are modeled as wire springs with zero original length and identical stiffness to that of the elastic sheet, whereas joint 3 is an ideal revolute joint. For the two-layer assembly, a cuboid with a thickness of 15 mm is used to connect the two units to avoid physical interference in the motion process. Solid contact is applied with setting Dry and penetration 0.001mm. During deformation, the P_{bot} is fixed and a linear or rotary motor is applied to the P_{top} to realize tension or twist. In addition, a loading rate of 0.2mm/s or 0.0115 rad/s is used to ensure a quasi-static process.

The single unit with μ = 0.706 and a = 80mm is first simulated, and the numerical results are presented together with the theoretical and experimental ones in Fig. 3E. A very good match is obtained, thus validating the theory and numerical model. Subsequently, the assemblies in Fig. 4 with different geometries and loading conditions are analyzed numerically using the same approach. Comparison of the theoretical, numerical, and experimental results are respectively presented in Fig. S8A-F. For each assembly, the numerically obtained transformation of stable states is identical to those obtained theoretically and experimentally. And the numerically predicted peak forces or torques at the transformation points match with the theoretical values very well. Therefore it can be concluded that the theoretical model can accurately predict the transformation process and required peak loads.

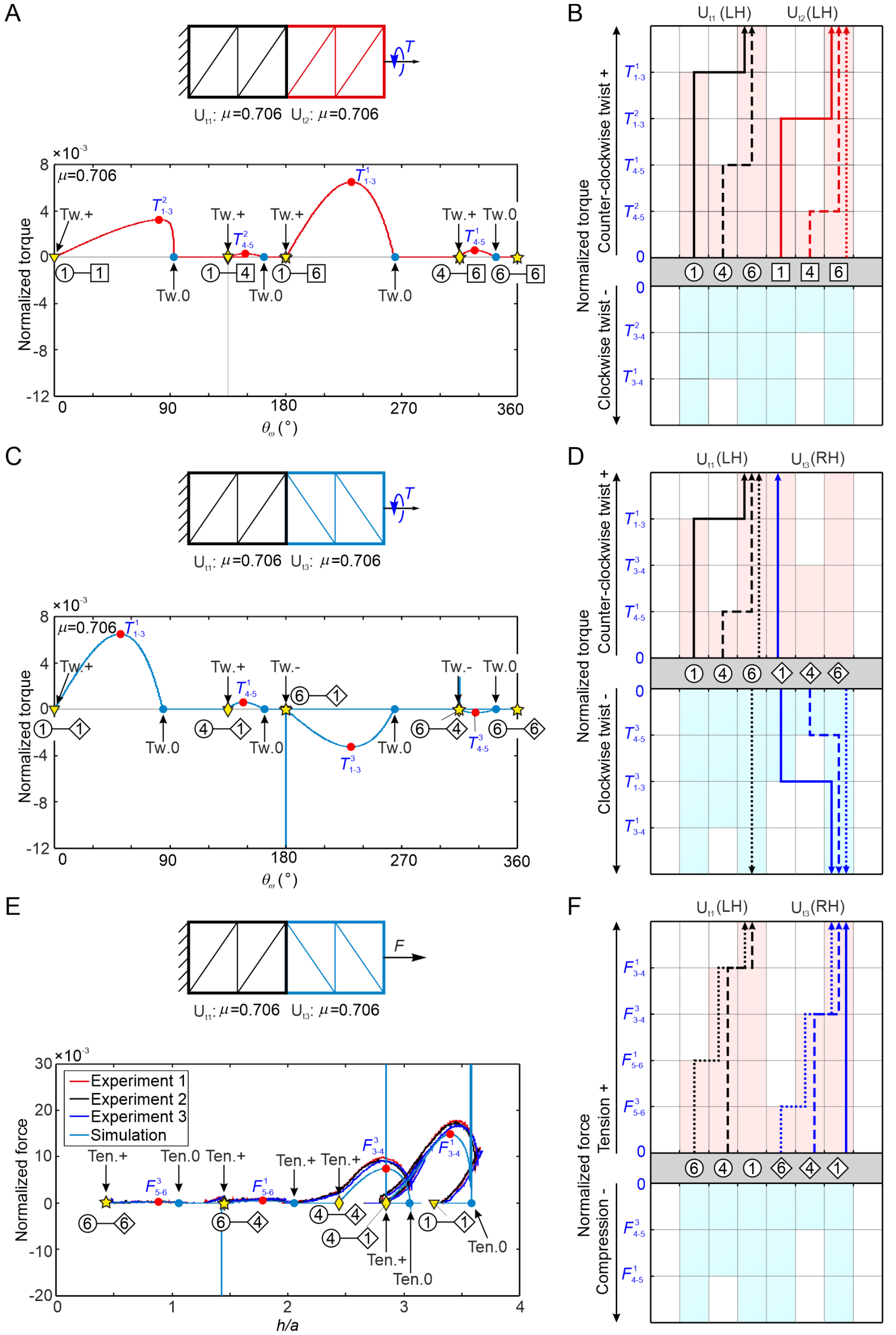

Fig. S8. The transformation of table states of assemblies with two tristable units corresponding to Fig.4. (A) The assembly of left-handed units U_{t1} and U_{t2} with $\mu = 0.706$ and $K_{e1,e5}^{Ut1} / K_{e1,e5}^{Ut2}$ $=K_{e2,e4}^{Ut1}/K_{e2,e4}^{Ut2}$=2, and the corresponding T *vs.* θ_ω curve. (B) The relationships between the stable

states and the regions of normalized torques during the transformation from stable state ①-1⃞ in the assembly. (C-D) the same set of figures as (A-B) for the assembly of U_{t1} (left-handed, μ = 0.706) and U_{t3} (right-handed, μ = 0.706) with $K^{\mathrm{Ut1}}_{\mathrm{e1,e5}}/K^{\mathrm{Ut3}}_{\mathrm{e1,e5}}$ =$K^{\mathrm{Ut1}}_{\mathrm{e2,e4}}/K^{\mathrm{Ut3}}_{\mathrm{e2,e4}}$=2 under twist. (E) The numerical and experimental results of the assembly of U_{t1} and U_{t3} under tension. (F) The relationships between the stable states and the regions of normalized forces during the transformation from stable state ⑥-6⃟ in the assembly. Here, Ten. 0 and Tw. 0 represent the positions of releasing the load.

10. The Tessellation of Thick-Panel Tristable Structures

A. The tessellation of two units with different length ratio

To discuss the variation of length ratio μ, we choose an assembly of two tristable units U_{t7} (right-handed, μ = 1) and U_{t1} (left-handed, μ = 0.706), where the thickness of latex film on the hinges are identical to 0.3mm. The schematic diagram of the assembly and curves of forces *vs. h/a* are shown in Fig. S9 which indicates $F^1_{4-5} < F^7_{4-5} < 0 < F^7_{5-6} < F^1_{5-6} < F^1_{3-4} < F^7_{3-4}$. Here, the transformation from stable states 6⃟-⑥ is analyzed with tension (Fig. S9B). Because the length ratio is different in the U_{t7} and U_{t1}, the energy barriers of the two units are different which leads to the transformation path in the order of states 6⃟-⑥, 4⃟-⑥, 4⃟-④, 4⃟-① and 1⃟-① (Fig. S9C, Movie S6). Here, by changing the length ratio μ, the stable state 4⃟-⑥ is obtained, which is different from the transformation path in Fig. 4I.

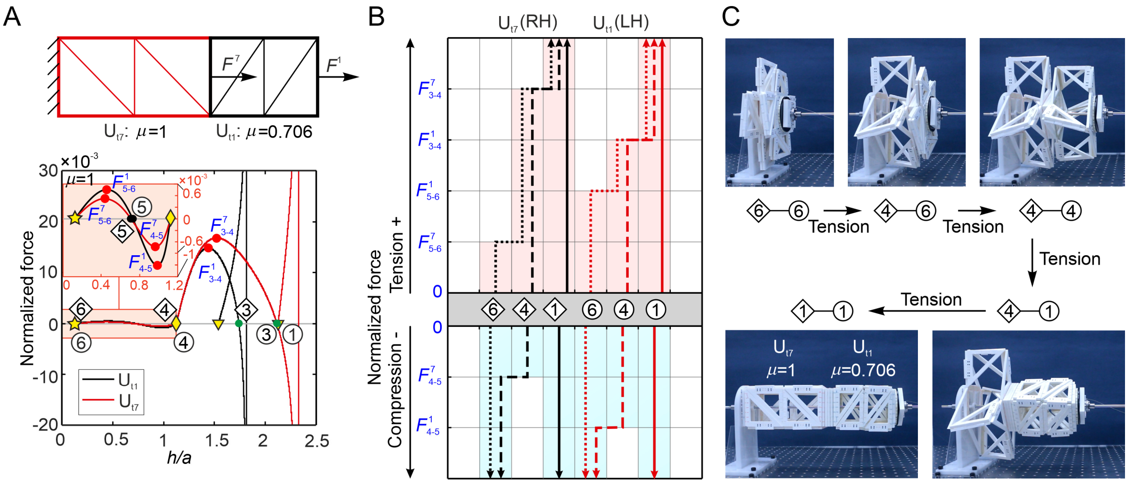

Fig. S9. The tessellation of tristable units to program the multiple stable states controlled by input tensions. (A) The assembly of tristable structures U_{t7} (right-handed, μ = 1) and U_{t1} (left-handed, μ = 0.706), and the corresponding curves of forces F^7 or F^1 *vs. h/a* of the assembly. (B) The relationships between the stable states and the regions of normalized forces during the transformation from an arbitrary stable state in the assembly. (C) The transformation of stable states for the assembly of U_{t7} and U_{t1}.

B. A programmed tessellation of three units with multiple stable states.

A multistable tessellation of three tristable units (left-handed U_{t4} and right-handed U_{t5} with $\mu = 0.706$, right-handed U_{t6} with $\mu = 1$) is carefully programmed through parameters in length ratio μ, elastic joint stiffness, cuboid chirality, loading modes, as shown in Fig. 5. For hinges 1 and 5, latex films with thickness 0.4 mm, 0.3 mm (cutting holes, see Fig. S5A), and 0.3 mm are used to U_{t4}, U_{t5}, U_{t6}, respectively. For hinges 2 and 4, films with thickness 0.3 mm, 0.4 mm, and 0.3 mm (cutting holes, see Fig. S5A) are used to U_{t4}, U_{t5}, U_{t6}, respectively. Then the stiffness in Fig. 5A is obtained, which determine the magnitude of critical forces/torques with length ratio μ according to SI appendix, section 7, and relationships between the stable states and the regions of normalized forces/torques during the transformation from an arbitrary stable state in the assembly are obtained, as shown in Fig. 5B. The transformation path of 12 stable states (Fig. 5C) is obtained by controlling input rotations or tensions (Movie S7). The setup of the rotation and tension process is the same as that shown in Fig. S7B. The fixed supporter is used to fix the left end of U_{t4} and the right supporter is used to support the specimen through the metal shaft to avoid the influence of gravity.

11. Frequency Reconfigurable Antenna

A. Design and fabrication

The antenna is designed using ANSYS High Frequency Structure Simulator (HFSS) according to the designed dimensions shown in Fig. S10A. The reconfigurable antenna includes two parts, antenna substrates with feeding network and radiator and a tristable metastructure, which is fed by an SMA connector for a coaxial cable. The FR4 with relative permittivity (εr) of 4.4, the thickness of 2 mm and loss tangent of 0.02 is selected as the antenna substrate. The antenna was fabricated by LPKF printed circuit broad (PCB) prototyping machine. The tristable metastructure was fabricated by Stratasys Dimension Elite 3D printer with ABS based on the dimensions in Fig. S10B, and the elastic sheet (length 24.2 mm and 50 mm, width 9 mm, thickness 0.3 mm) was cut by a Trotec Speedy 300 laser cutter. The assembly of the tristable metastructure is similar to that in Fig. S5B. The antenna and tristable metastructure are glued by 3M VHB double sticky tape.

B. Simulation

The antenna is simulated in HFSS. During the simulation, an airbox is employed and all six faces of the airbox are set as radiation boundaries. The distance between the antenna and radiation boundaries is around half wavelength at each resonant frequency, as shown in Fig. S10C.

C. Experiment

The setup of the electromagnetic experiment is shown in Fig. S10D. The antenna was measured in a near-field antenna pattern measurement chamber and connected to Rohde & Schwarz ZNA24 by the coaxial cable (Fig. S10E). By changing the state of the specimen and connecting the adjacent radiators with copper tape (3M 1181), frequencies can be measured and results are shown in Fig. 6D. Experiment and simulation radiation patterns at *xz*- and *yz*-planes of the three stable states resonant frequencies are exhibited in Fig. S10F. Measurement agrees with the simulations well.

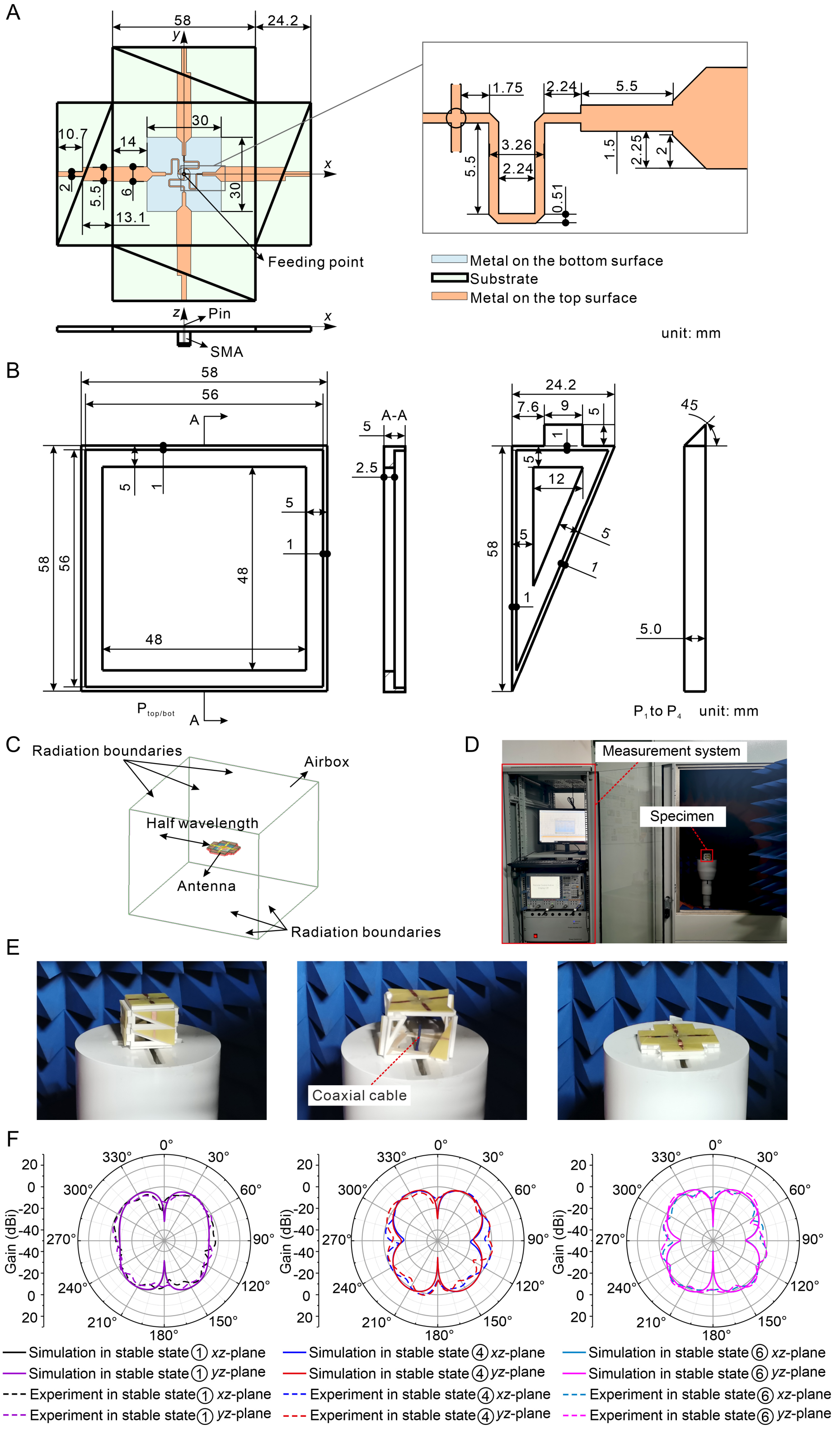

Fig. S10. Frequency reconfigurable antenna. (A) The dimensions of the antenna. (B) The dimensions of parts of the tristable metastructure. (C) The setup of simulation. (D) The setup of the experiment. (E) The three stable states and the connection between the antenna and the

measurement system. (F) Experiment and simulation radiation patterns at *xz*- and *yz*-planes of the three stable states resonant frequencies.

12. Stiffness of The Thick-Panel Tristable Structures

The stiffness of a single unit can be obtained by calculating the derivatives of Eq. S50 about force, as

$$K_F = \begin{cases} \dfrac{dF_{\mathrm{e2,e4}}}{dh} = \dfrac{dF_{\mathrm{e2,e4}} / d\varphi_2}{dh / d\varphi_2}, \text{ between states ① and ④} \\ \dfrac{dF_{\mathrm{e1,e5}}}{dh} = \dfrac{dF_{\mathrm{e1,e5}} / d\varphi_1}{dh / d\varphi_1}, \text{ between states ④ and ⑥} \end{cases}. \tag{S52}$$

Notice that only tension stiffness is considered at states ① and ⑥ as the unit is folded at those two configurations. For stable states ④, the tension stiffness is different from the compressive one due to the different deformation paths.

Based on the Eq. S52 (codes 'F3BE_DerivationsForForceTorqueEq_StableStates14.nb' and 'F3BE_DerivationsForForceTorqueEq_StableStates46.nb' in Dataset S3), the stiffness of the unit is dependent on the unit geometry ratio μ and joint stiffness. As shown in Fig. S11A-D, the tension stiffness at stable states ① and ④ increases with μ, whereas the tension stiffness at ⑥ and the compressive stiffness at ④ reduce with μ. In contrast, an increase in joint stiffness leads to a higher stiffness at all the stable states.

When a number of units are connected in series, the stiffness of the assembly at each stable state can be obtained as

$$\frac{1}{K_{\mathrm{ass}}} = \frac{1}{K_{\mathrm{Ut1}}} + \frac{1}{K_{\mathrm{Ut2}}} + \cdots + \frac{1}{K_{\mathrm{Ut}i}} + \cdots \tag{S53}$$

in which $K_{\mathrm{Ut}i}$ is the stiffness of the unit Ut_i (i = 1, 2, …).

Therefore, by adjusting the geometric and material parameters of each unit as well as the number of units, the stiffness of the assembly at each stable state can be programmed. To demonstrate this, the stiffnesses of all the stable states for the assembly in Fig. 4I are calculated and presented in Fig. S11E. The two units in the assembly have identical geometry but different joint stiffness, leading to different stiffnesses at different stable states.

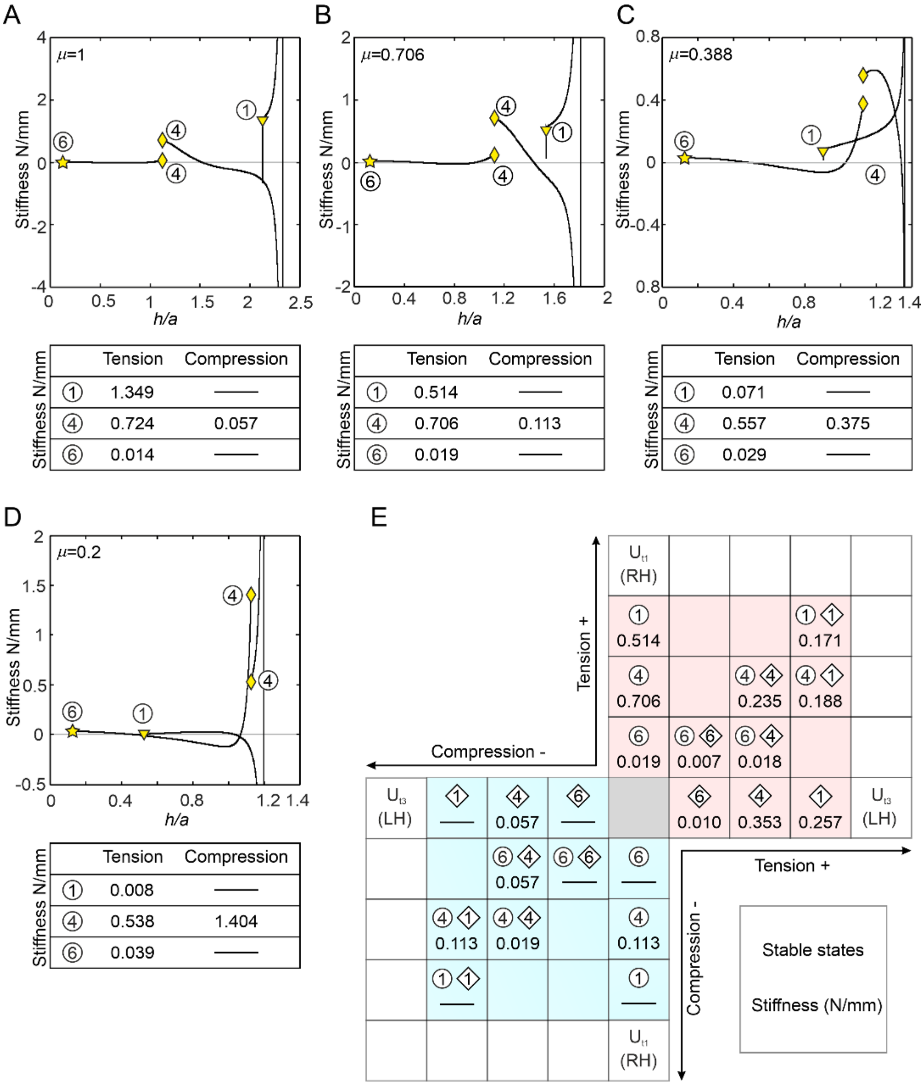

Fig. S11. The programmable stiffnesses of the tristable structure and its assembly. (A-D) The stiffness *vs.* *h/a* curve of the units with μ = 1, 0.706, 0.388 and 0.2, and the corresponding stiffnesses at stable states. (E) The stiffnesses for all the stable states of the assembly in Fig. 4I.

Movie S1. Folding sequence of the right-handed kirigami cuboid.

Movie S2. Kirigami cuboid with torsional springs in Fig. 2A to Fig.2C.

Movie S3. The wood tristable structures with μ = 1 and 0.388.

Movie S4. The tension experiment of a tristable structure with μ = 0.706.

Movie S5. Transformation path of stable states about assemblies of two units under twist in Fig. 4C and Fig. 4F.

Movie S6. Transformation path of stable states about assemblies of two units under tension in Fig. 4I and Fig. S9C.

Movie S7. Transformation path of stable states about the assembly of three units.

Dataset S1. The data about the experiment of an elastic joint in Fig. S2D

Dataset S2. Multistable_structure_Datas_0.706

The data about the experiment and simulation of multistable structures with μ = 0.706 in Fig. 3E, Fig. S8.

Dataset S3. Functional codes for the derivations of equations and curves in figures

Dataset S4. Antenna_Datas

The data about the experiment and simulation of the antenna in Fig. 6.

References

1. F. Yang, Y. Chen, R. Kang, J. Ma, Truss Transformation Method to Obtain the Non-Overconstrained Forms of 3d Overconstrained Linkages. *Mechanism and Machine Theory* **102**, 149-166 (2016).
2. J. Denavit, A kinematic notation for lower-pair mechanisms based on matrices. *Journal of Applied Mechanics* **22**, 215-221 (1955).
3. SolidWorks, D S. SolidWorks®. Version SolidWorks (2005).
